# Diterpenoid Phytoalexins Shape Rice Root Microbiomes and Their Associations With Root Parasitic Nematodes

**DOI:** 10.1111/1462-2920.70084

**Published:** 2025-03-28

**Authors:** Enoch Narh Kudjordjie, Willem Desmedt, Tina Kyndt, Mogens Nicolaisen, Reuben J. Peters, Mette Vestergård

**Affiliations:** ^1^ Department of Agroecology, Faculty of Technical Sciences Aarhus University Slagelse Denmark; ^2^ VIB Center for Plant Systems Biology Ghent Belgium; ^3^ Research Group Epigenetics and Defence, Department of Biotechnology Ghent University Ghent Belgium; ^4^ Department of Plant Biotechnology and Bioinformatics Ghent University Ghent Belgium; ^5^ Roy J. Carver Department of Biochemistry, Biophysics & Molecular Biology Iowa State University Ames Iowa USA

**Keywords:** diterpenoid mutation, *Meloidogyne graminicola*, rhizosphere/root microbiome, rice mutants, root‐knot nematodes, secondary metabolites

## Abstract

Rice synthesises diterpenoid phytoalexins (DPs) which are known to operate in defence against foliar microbial pathogens and the root‐knot nematode *Meloidogyne graminicola*. Here, we examined the role of DPs in shaping rice‐associated root microbiomes in nematode‐infested field soil. Further, we assessed how DPs affect interactions between the root microbiomes and *M. graminicola* . We used 16S and ITS2 rRNA gene amplicon analysis to characterise the root‐ and rhizosphere‐associated microbiomes of DP knock‐out rice mutants and their wild‐type parental line, at an early (17 days) and late (28 days) stage of plant development in field soil. Disruption of DP synthesis resulted in distinct changes in the composition and structure of microbial communities both relative to the parental/wild‐type line but also between individual mutants, indicating specificity in DP‐microbe interactions. Moreover, the abundance of nematode‐suppressive microbial taxa, including *Streptomyces*, *Stenotrophomonas* and *Enterobacter* was negatively correlated with that of *Meloidogyne*. Differential enrichment of microbial taxa in the roots of rice DP knock‐out mutants versus wild‐type suggests that DPs modulate specific taxa in the rice root microbiome. These findings indicate a role for DPs in plant‐microbiome assembly and nematode interactions, further underscoring the potential of leveraging phytoalexins for sustainable management of crop diseases.

## Graphical abstract

Diterpenoid phytoalexins (DPs) defend plants against microbial pathogens and parasitic nematodes. This study examined the role of DPs in shaping rice‐associated microbiomes, using DP‐disrupted mutants, and provided insights into DP‐modulating activities of microbiomes, pathogens and root‐knot nematodes, offering a new frontier in breeding DP‐enhanced crops for disease resistance.

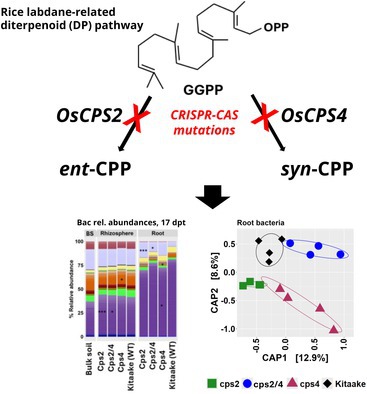

## 1 Introduction

Plants must constantly defend themselves against biotic and abiotic stressors. To protect themselves against microbial pathogens or parasitic nematodes, plants as sessile organisms have adapted constitutive (i.e., always present) and inducible (i.e., stress‐induced) chemical repertoires that are part of the conserved immune system (Chisholm et al. 2006). Inducible defence responses which are triggered by stressors including pathogens are mediated in part by stress‐inducible secondary metabolites collectively named phytoalexins. Phytoalexins are a structurally diverse class of low weight molecules produced by the host in response to biotic and abiotic stressors (VanEtten et al. 1994). Several classes of phytoalexins, including terpenoids (diterpenoids, triterpenoids, sesquiterpenoids), phenylpropanoids and benzoxazinoids, have been identified in cereals, with profound protective roles against microbial pathogens, nematodes and abiotic stresses (Dixon 2001; Lu et al. 2018; Sikder et al. 2021; Desmedt et al. 2022; Kaur et al. 2022). The ability of phytoalexins to defend the host against pathogens is being investigated as a potential target for the sustainable control of plant diseases (Jeandet et al. 2017).

Diterpenoids are among the major classes of phytoalexins produced in several monocots including rice ( *Oryza sativa* ) (Yamane 2013; Murphy and Zerbe 2020), in which the predominant phytoalexins include the diterpenoid momilactones, phytocassanes and abietoryzins (Schmelz et al. 2014; Kariya et al. 2020, 2023). Biosynthesis of the rice diterpenoid phytoalexins (DPs) proceeds via either *ent*‐ or *syn*‐copalyl diphosphate (CDP) intermediates produced from the general diterpenoid precursor geranylgeranyl diphosphate (GGDP) (Yamane 2013; Schmelz et al. 2014). GGDP undergoes cyclization to form either *ent*‐CDP or *syn*‐CDP, catalysed by CDP synthases (CPSs), with *syn*‐CDP exclusively produced by OsCPS4, and *ent*‐CDP produced by OsCPS1 and OsCPS2 (Otomo et al. 2004; Prisic et al. 2004; Xu et al. 2004). OsCPS1 is specifically required for gibberellin phytohormone biosynthesis (Sakamoto et al. 2004), while the expression of OsCPS2 is inducible and associated with DP biosynthesis (Toyomasu et al. 2008). Subsequently, rice kaurene synthase‐like (OsKSL) enzymes convert *syn*‐CDP or *ent*‐CDP into olefin precursors of the various groups of DPs (Yamane 2013; Schmelz et al. 2014).

Rice DPs are produced in different plant organs and accumulate during stress conditions such as exposure to UV or pathogen attack (Yamane 2013; Schmelz et al. 2014). Rice roots exude DPs such as momilactones and phytocassanes into the rhizosphere (Toyomasu et al. 2008). These DPs have a broad spectrum of biocidal activities against microbial pathogens such as *Magnaporthe oryzae* and *Xanthomonas oryzae* (Toyomasu et al. 2008). In addition, terpenoid phytoalexins in other plants have been shown to modulate the assembly of their associated microbiomes (Huang et al. 2019; Murphy et al. 2021). For example, Li et al. (2023) reported differential inhibitory effects of distinct switchgrass DPs on fungal taxa in their rhizosphere, and Murphy et al. (2021) recently showed that maize‐secreted DPs affect bacterial communities in the rhizosphere.

Our previous work with rice DP‐deficient mutant lines grown in field soil demonstrated that these secondary metabolites protect rice plants against the phytoparasitic root‐knot nematode *Meloidogyne graminicola* , likely through a combination of direct nematostatic activity and alterations in root‐associated nematode communities that include the attraction of predatory nematodes believed to feed on phytoparasitic nematodes (Desmedt et al. 2022). In addition to the direct effects of DPs on root nematodes, these secondary metabolites may modulate the root microbiome in ways that alter susceptibility to nematode pests. Root microbiomes assembled by other plants during pathogen or pest attack have been shown to encompass beneficial microbes that are able to suppress plant parasitic nematodes through the secretion of secondary metabolites and lytic enzymes (Chinheya et al. 2017; Poveda et al. 2020). Examples include rhizobacteria such as *Bacillus* spp., *Rhizobium* spp., *Burkholderia* spp., *Pseudomonas* spp. and the fungi *Trichoderma* spp. and *Clonostachys rosea* (Chinheya et al. 2017; Iqbal et al. 2018; Poveda et al. 2020). Therefore, in this study, we sought to examine the effect of DPs on rice‐associated microbiomes assembled in *M. graminicola* – infested field soil.

We hypothesise that DPs not only increase rice resistance towards *M. graminicola* by directly affecting nematodes but also act through effects on the root‐associated microbiomes. We therefore determine the effects of rice DPs by using DP disruption mutants at different growth stages and *M. graminicola* infection. We analysed the root microbiome and examined the co‐occurrence of various microbiota and nematodes, specifically focusing on *M. graminicola* .

## 2 Materials and Methods

### 2.1 Plant Material and *M. graminicola* Culture

We used the rice wild‐type (WT) *O. sativa* L. subsp. *japonica* cv. Kitaake and DP‐deficient CRISPR‐Cas9 knock‐out mutants disrupted in *OsCPS2* (*cps2*), *OsCPS4* (*cps4*) and the double mutant *cps2/4*, in the Kitaake genetic background. In *cps2*, the accumulation of *ent*‐CPP DPs is significantly reduced, and in *cps4*, the production of *syn*‐CPP DPs is blocked. The DP profiles of this parental/wild‐type and these mutants, as well as their lack of effect on plant growth and development, have been previously described (Zhang et al. 2021).

*M. graminicola* was originally isolated in Batangas, the Philippines (kindly provided by Prof. Dirk De Waele) and cultured on susceptible *Echinochloa cruss‐galli* plants in a growth room at 28°C. Second‐stage juveniles (J2s) were extracted using a sieve/tray extraction method. The taxonomic identity was confirmed using sequencing of the ITS region.

### 2.2 Experimental Design and Sampling

Experimental design, growing of plants, sample collection and DNA extraction from rice lines was previously described (Desmedt et al. 2022). Briefly, rice lines were grown in pots filled with 250 mL of naturally *M. graminicola* infested field soil with a long history of rice cultivation, collected on 26 August 2021 in the commune of Garlasco (Pavia province, Lombardy, Italy). Each pot was planted with one rice seedling (5 days after placing the seed on wet tissue paper), representing a biological replicate. Seedlings were placed in a greenhouse at 26°C and 12 h of artificial light per day for the entire duration of the experiment. During the first 24 h after transplantation to the pots, the seedlings were covered with a transparent plastic sheet. The pots were irrigated with 70 mL of distilled water at transplantation and then with 15 mL distilled water daily. We fertilised the pots twice, at 1 and 14 days post transplantation (dpt), with 15 mL of a fertiliser solution containing 2 g L^−1^ ferrous sulfate heptahydrate and 1 g L^−1^ ammonium sulfate. Seven days after transplantation, we inoculated the soils with an additional 120 *M. graminicola* infective stage J2s, as described in Desmedt et al. (2022), to ensure a high probability of root infestation.

We sampled bulk soil, rhizosphere soil and roots at two time points, at 17 and 28 dpt, as described previously (Desmedt et al. 2022). These two time points were chosen to represent key stages in the root‐knot nematodes' life cycle; 17 days after sowing corresponds to 5 days after the supplemental inoculation with *M. graminicola* , a time point coinciding with peak gall and feeding site formation. Similarly, 28 days after sowing (16 days after supplemental inoculation) was chosen to represent the start of egg‐laying. We carefully uprooted five seedlings from each line and gently shook the roots to remove nonadherent soil, after which adhering rhizosphere soil was gently scraped off and flash‐frozen in liquid nitrogen. We then washed the root systems thoroughly with distilled water and froze the roots separately. The collected samples were kept frozen, lyophilized for 48 h, and prepared for DNA extraction by grinding with sterile steel beads at 3 × 1500 strokes per min in a GenoGrinder (Ramcon, Birkerød, Denmark). We extracted DNA from soil samples using a Qiagen PowerLyzer Soil kit, and from root samples using a Qiagen DNeasy Plant Pro kit. DNA concentrations were checked using a Qubit fluorometer (Thermo‐Fisher Scientific, Waltham, MA, USA), prior to downstream processing.

### 2.3 Amplicon Library Preparation

To characterise microbial communities, we amplified and sequenced bacterial 16S rDNA and fungal ITS regions by following a previously described procedure (Kudjordjie et al. 2019). The microbiome metabarcoding was done with the same DNA samples used for nematode community profiling in Desmedt et al. (2022). Briefly, bacterial amplicon libraries were constructed by amplifying 16S rRNA V5–V7 with 799F/1193R primer pairs (Chelius and Triplett 2001; Bodenhausen et al. 2013; Beckers et al. 2016). Bacterial amplification was performed in a 25 μL reaction mix consisting of 12.5 μL Invitrogen Platinum SuperFi PCR master mix (Thermo Fisher Scientific, Waltham, Massachusetts), 2 μL of each primer (10 μM stock), and 6.5 μL nuclease‐free water and 2 μL of the template DNA. PCR amplification was performed in a GeneAmp PCR System 9700 thermal cycler (Thermo Fisher Scientific) at the following conditions: 94°C for 5 min; 25 cycles at 94°C for 30 s, 55°C for 30 s and 72°C for 30 s; and a final extension step at 72°C for 10 min. The fungal library was prepared using the fITS7 and ITS4 fungal primer pair that amplify the internal transcribed spacer 2 (ITS2) region of the fungal rRNA gene (Ihrmark et al. 2012). Amplification was done in a reaction mixture of 25 μL consisting of 1× PCR reaction buffer, 1.5 mM MgCl_2_, 0.2 mM dNTPs, 1 μM of each primer, 1 U of GoTaq Flexi polymerase (Promega Corporation, Madison, USA) and 1 μL of DNA template. PCR thermal cycling conditions for fungal PCR were as described above, except for an annealing temperature of 57°C (Ihrmark et al. 2012). We used dual indexing in combination with internal barcodes to pool samples. The bacterial and fungal forward primers were tagged with varying bases of multiplex identifiers (MID). For indexing, primers including indexing tags were used in a PCR for 10 cycles, with the thermal cycler programs as described above. The index combinations and fungal primer sequences used are described in Kudjordjie et al. (2019), while bacterial primer sequences including MID are presented in Table S1. After PCR, amplicon sizes of both bacterial and fungal libraries were confirmed on a 1.5% agarose gel, and bands of the expected sizes were excised and extracted using QIAquick Gel Extraction Kit (Qiagen). The libraries were sequenced on an Illumina MiSeq sequencer with PE300 at Eurofins MWG (Ebersberg, Germany).

### 2.4 Sequence Data and Statistical Analysis

Bacterial and fungal sequence reads were analysed as described earlier (Kudjordjie et al. 2019). Briefly, paired‐end reads were demultiplexed for internal barcodes using Mr_Demuxy with the command pe_demuxer.py (https://pypi.org/project/Mr_Demuxy/). Following this, the paired‐end reads were assembled and joined using vsearch v.2.6 (Rognes et al. 2016). Primers were removed using cutadapt (Martin 2011), followed by dereplication, chimaera removal and operational taxonomic unit (OTU) clustering using vsearch v.2.6 (Rognes et al. 2016). Fungal ITS reads were extracted with ITSx extractor version 1.0.6 (Bengtsson‐Palme et al. 2013) before clustering. Taxonomic assignments of bacterial and fungal OTUs were performed using the SILVA 132 (Quast et al. 2013) and the UNITE (v7.2) (Abarenkov et al. 2010) databases, respectively, with assign_taxonomy.py in QIIME (v1.9) (Caporaso et al. 2010). The unassigned OTUs at the kingdom level or OTUs assigned as chloroplast or mitochondrial sequences were removed.

Microbial analysis and visualisations were performed in R v4.0.5 (R Core Team 2022), using phyloseq (v1.34.0.) (McMurdie et al. 2013), vegan (v2.5.7) (Oksanen et al. 2020) and ggplot2 (v3.3.2) (Wickham 2009) packages. Sequences with less than 2000 and 1000 reads were removed from the bacterial and fungal data sets, respectively. Differences in the microbial relative abundances between mutants and wild‐type was determined using the Wilcoxon test and *p*‐values were adjusted with the false discovery rate correction for multiple comparisons (fdr) using the compare_means() function in the R package ggpubr (v0.6.0) (https://rpkgs.datanovia.com/ggpubr/). For alpha diversity, OTU tables were rarified 100 times at a depth of 2000 reads for bacteria and 1000 reads for fungi and the mean of the diversity estimates of 100 trials was used to estimate observed species richness and Shannon diversity. Significant differences between alpha diversities were calculated using the Kruskal‐Wallis rank sum test. Before beta diversity analysis, OTU tables were transformed into relative abundances. The Bray–Curtis dissimilarity matrix was calculated to construct both unconstrained principal coordinates analysis (PCoA) and constrained principal coordinate (CAP) visualisations. CAP analysis was constrained by the host genotype using the function ‘ordinate’ in the phyloseq package. Permutational analysis of variance (PERMANOVA) was computed on both bacterial and fungal datasets to quantify differences between experimental factors by using the adonis2 function from the ‘vegan’ package (Oksanen et al. 2020). The analysis was performed using subset datasets of compartment (root, rhizosphere) and time points 17 and 28 dpt. Further pairwise comparisons between WT and individual mutants were performed using the pairwise.adonis2 function (https://github.com/pmartinezarbizu/pairwiseAdonis/blob/master/pairwiseAdonis/R/pairwise.adonis2.R). Relative abundances of *M. graminicola* in rice mutants and WT were also computed to support nematode colonisation of rice plants.

Microbial co‐occurrence analysis was performed using a previously described procedure (Kudjordjie et al. 2019). Briefly, bacterial, fungal and nematode datasets (Desmedt et al. 2022) from 17 and 28 dpt were pooled, subjected to trimmed mean of M values (TMM) procedure using EdgeR (Robinson et al. 2010) in R. TMM‐normalised data were used to calculate Spearman rank correlations between microbial OTUs using the corr function. We used OTUs that were present in at least 20% of the samples with *r* > 0.4 for positive correlations and *r* < −0.4 for negative correlations, and *p*‐values < 0.01. The correlations were visualised in heatmaps.

For individual bacterial and fungal taxa, differential abundance analysis between WT and individual mutants was performed using ANCOM‐BC (Lin and Peddada 2020). ANCOM‐BC utilises linear regression and controlled FDR to identify differentially abundant taxa between rice mutants and their parental wild type.

## 3 Results

To establish whether and how DPs modulate root‐associated microbiomes in rice, we compared the bacterial and fungal communities of the WT and derived DP‐disrupted mutants. Bacterial 16S and fungal ITS regions were amplified and sequenced; the bacterial amplicon library yielded a total of 430,237 high‐quality sequences (range 3004– 9053; median 5975) resulting in 3534 bacterial OTUs in 77 samples, while the fungal library produced 1,424,701 sequences (range 2391—83,684; median 11,659) resulting in 772 OTUs in 90 samples. We provide detailed sequence read statistics in Table S2 and the read distribution and rarefaction curves are given in Figure S1. The rarefaction curves were reaching an asymptote, reflecting satisfactory representation of microbial taxa in the samples. Because microbial data was obtained from *M. graminicola* infested samples, we correlated the microbial data with the nematode sequence reads obtained from our previous study, which showed that DPs play a role in rice defence against root‐knot nematodes (Desmedt et al. 2022). Nematode sequence reads assigned to the genus *Meloidogyne* accounted for > 90% of reads (WT: 90%, cps2: 96%, cps4: 96%, cps2/4: 93%; *p* = 0.61), with higher relative abundances in roots at 17 dpt than 28 dpt. However, at the latter time point, *cps* mutants had significantly higher abundances of *Meloidogyne* than the wild‐type (Desmedt et al. 2022).

### 3.1 Diterpenoids Shape Root and Rhizosphere Microbiomes

**FIGURE 1:**
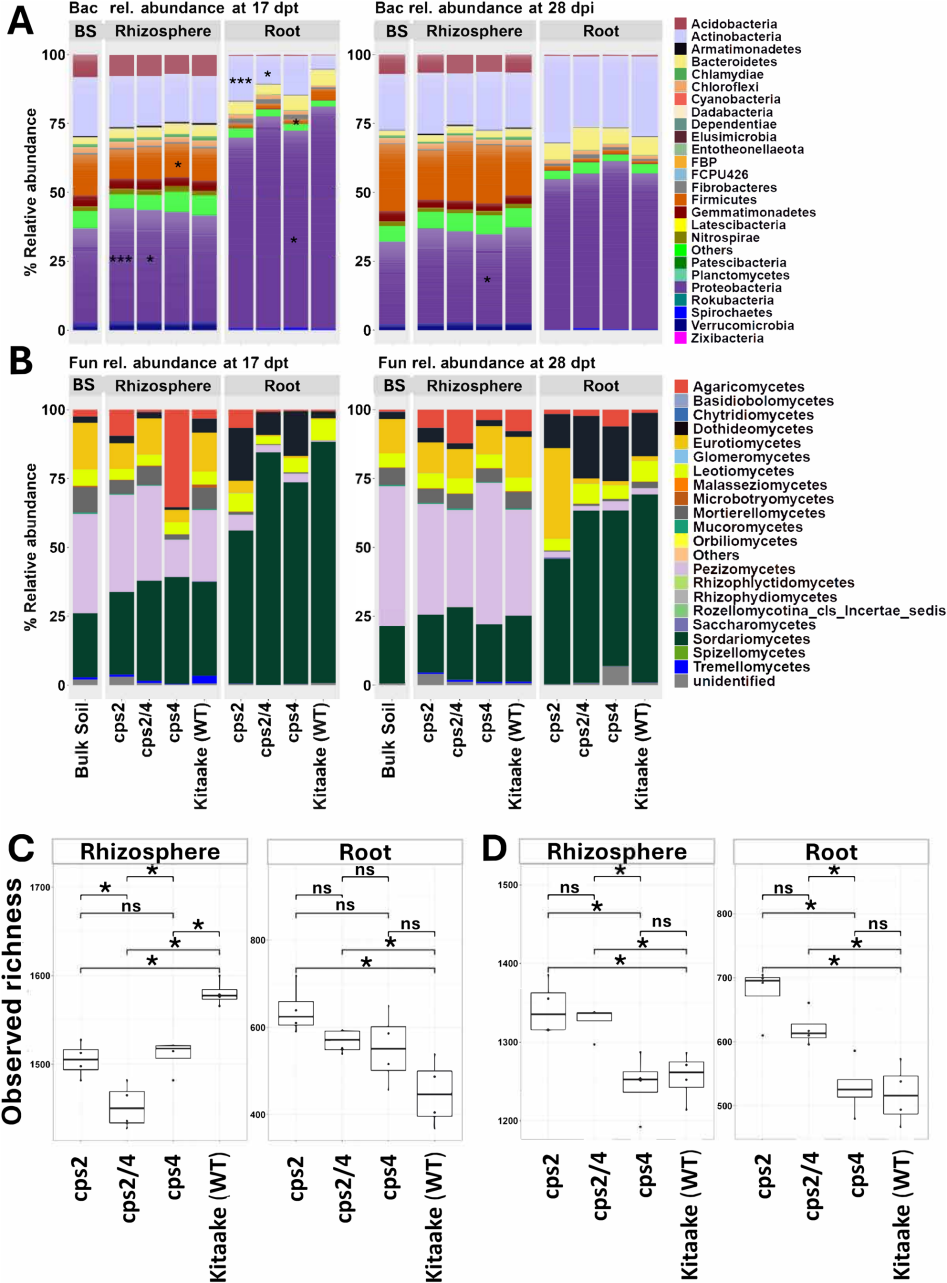
Microbial taxonomic profiles in different rice compartments and growth stages. (A) Relative abundances of bacterial taxa (phyla‐level) in root, rhizosphere and bulk soil at 17 and 28 dpt. (B) Relative abundances of fungal taxa (class‐level) in root, rhizosphere and bulk soil at 17 and 28 dpt. Bacterial observed richness in wild‐type and mutant rice lines in the rhizosphere and root compartments at (C) 17 and (D) 28 dpt. Microbial taxa with significantly different relative abundances between mutants and wild‐type Kitaake are indicated in asterisk in the bar plots (*p < 0.05; **p < 0.01 and ***, *p* < 0.001). Comparisons were performed using Wilcoxon test and *p*‐values were adjusted for multiple comparisons (fdr) using the compare_means() function in the R package ggpubr (v0.6.0).

We found marked differences in the abundances of bacterial phyla and fungal classes in the roots of DP disruption mutants compared to WT (Figure 1A,B). At 17 dpt, the relative abundances of bacterial phyla Proteobacteria were significantly higher in the rhizosphere of *cps4* and *cps2/4* compared with WT. Similarly, Firmicutes were higher in *cps4* than WT. In roots, the abundance of Actinobacteria was higher in the mutants than in WT at 17 dpt. Actinobacteria were highly enriched in *cps2* and *cps2/4*, while Firmicutes and Proteobacteria significantly decreased in *cps4* compared with WT. Also, Proteobacteria was lower in *cps4* at 28 dpt. The relative abundances of specific fungal classes were comparable in the roots and rhizosphere of all genotypes (Figure 1B).

At the OTU level, bacterial richness in the rhizosphere was lower in all the mutants compared to WT at 17 dpt (Figure 1C), whereas bacterial richness was higher in the *cps2* and *cps2/4* mutants compared to WT in the roots at both sampling times and in the rhizosphere at 28 dpt (Figure 1D). Bacterial richness was lowest in the rhizosphere of *cps2/4* at 17 dpt (Figure 1C) and significantly higher in the rhizosphere and roots of *cps2* than in *cps4* at 28 dpt (Figure 1D). Fungal OTU richness was less responsive to the mutations, but at 17 dpt *cps2* roots had higher fungal richness than WT (Figure S2). Moreover, the composition of microbial communities was more distinct in the roots compared with the bulk soil and rhizosphere compartments, with strong effects exerted by both genotype and compartment on bacterial and fungal communities (Figures S3 and S4, Table S3).

**FIGURE 2:**
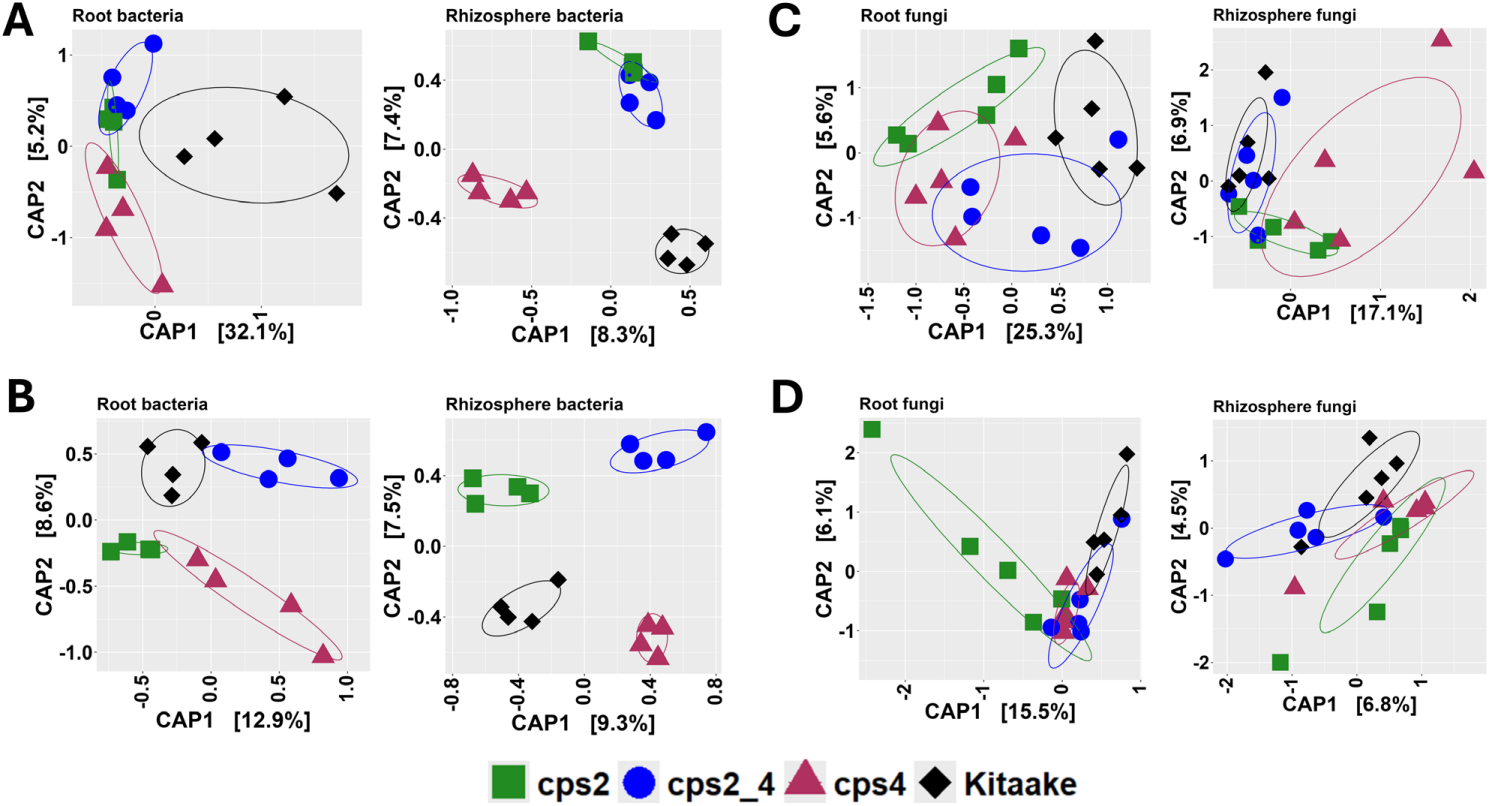
Host genotype effects on microbial communities in the root and rhizosphere compartments. Constrained analysis of principal coordinates (CAP) of bacterial communities in rice root and rhizosphere at (A) 17 dpt and (B) 28 dpt and similarly for fungal communities at (C) 17 dpt and (D) 28 dpt using Bray–Curtis dissimilarity distances.

Similarly, at 17 dpt, the root bacterial community composition assessed at OTU level differed significantly between all genotypes. In the rhizosphere, bacterial communities in mutants were all significantly different compared to WT. Also, bacterial communities in the *cps4* mutant were significantly different from *cps2* and *cps2/4* (Figures 2A and S4, Tables 1 and S4). At 28 dpt, differences between bacterial communities in roots from WT and mutants were not significant (Figure 2B, Table 1). Among the mutants, the rhizosphere bacterial communities of *cps2* were significantly different from *cps4* (Adonis, R^2^ = 0.18, *p* < 0.05) and *cps2/4* (Adonis, R^2^ = 0.18, *p* < 0.05) at 28 dpt (Table S4). For the fungi, the community composition of the WT was significantly different from both the single mutant lines (*cps2* and *cps4*) in both roots and rhizosphere at 17 dpt and in roots at 28 dpt (Figure 2C,D, Table 1). Root fungal communities were also significantly different between *cps2* and *cps2/4* (Table S4).

**TABLE 1:** PERMANOVA (Pairwise‐adonis) between rice mutants and wild‐type (Kitaake).

Community	DPI	Compartment	Factors	*R* ^2^
Bacteria	17	Root	cps2_vs_Kitaake	0.38*
			cps4_vs_Kitaake	0.36*
			cps2/4_vs_Kitaake	0.35*
		Rhizosphere	cps2_vs_Kitaake	0.16 ns
			cps4_vs_Kitaake	0.18*
			cps2/4_vs_Kitaake	0.15 ns
	28	Root	cps2_vs_Kitaake	0.17 ns
			cps4_vs_Kitaake	0.19 ns
			cps2/4_vs_Kitaake	0.19 ns
		Rhizosphere	cps2_vs_Kitaake	0.176 ns
			cps4_vs_Kitaake	0.15 ns
			cps2/4_vs_Kitaake	0.17 ns
Fungal	17	Root	cps2_vs_Kitaake	0.38*
			cps4_vs_Kitaake	0.37**
			cps2/4_vs_Kitaake	0.19 ns
		Rhizosphere	cps2_vs_Kitaake	0.29*
			cps4_vs_Kitaake	0.22*
			cps2/4_vs_Kitaake	0.09 ns
	28	Root	cps2_vs_Kitaake	0.22**
			cps4_vs_Kitaake	0.20*
			cps2/4_vs_Kitaake	0.16 ns
		Rhizosphere	cps2_vs_Kitaake	0.09 ns
			cps4_vs_Kitaake	0.10 ns
			cps2/4_vs_Kitaake	0.12 ns

*Note:* Significance of test indicated as **p < 0.01; *p < 0.05. The ns denotes not statistically significant and *R* ^2^ is the proportion of variation explained.

### 3.2 Diterpenoids Modulate the Relative Abundance of Several Bacterial and Fungal OTUs

**FIGURE 3:**
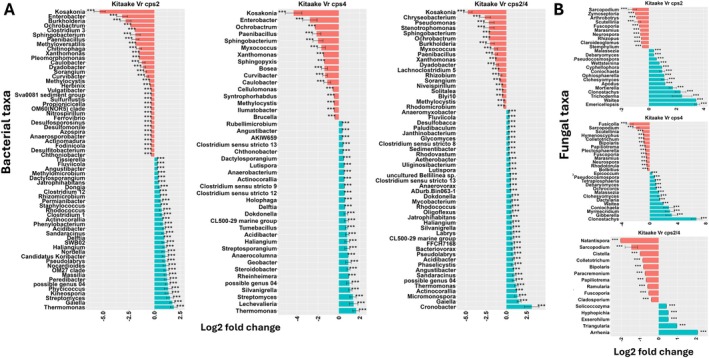
Differentiall abundant (log2 fold change) (A) bacterial and (B) fungal genera between roots of rice wild‐type and roots of individual mutants at 17 dpt. Data are represented by log fold change (shown as a column), ±SE (shown as error bars) derived from the ANCOM‐BC model. ANCOM‐BC implements Bonferroni correction to adjust for multiple comparisons. The red and green bars represent taxa that are enriched in Kitaake and mutants, respectively. The significance of test is indicated as ***p < 0.001, **p < 0.01 and *p < 0.05.

**FIGURE 4:**
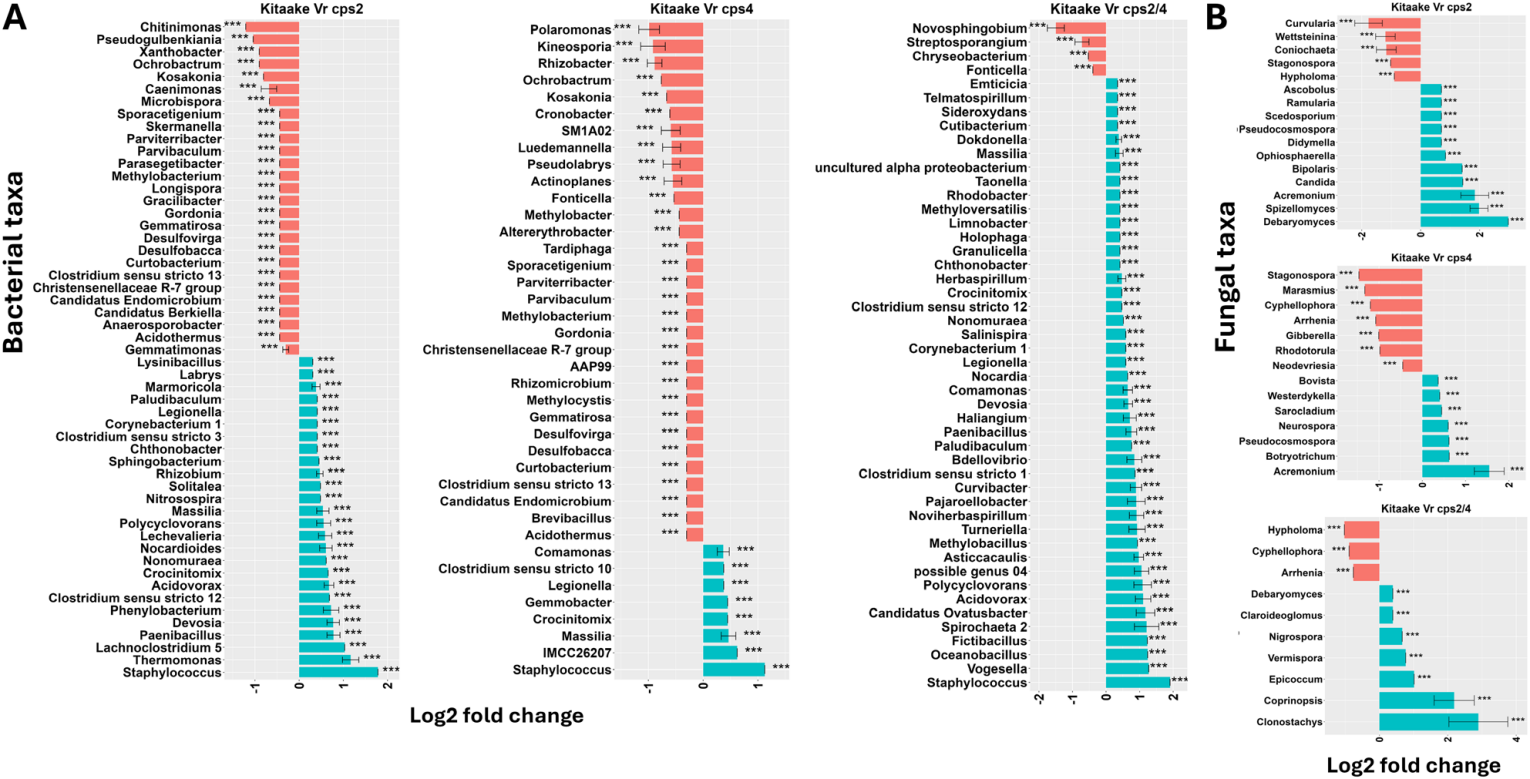
Differentially abundant (log2 fold change) (A) bacterial and (B) fungal genera between roots of rice wild‐type and roots of individual mutants at 28 dpt. Data are represented by log fold change (shown as a column), ±SE (shown as error bars) derived from the ANCOM‐BC model. ANCOM‐BC implements Bonferroni correction to adjust for multiple comparisons. The red and green bars represent taxa that are enriched in Kitaake and mutants, respectively. The significance of test is indicated as ***p < 0.001, **p < 0.01 and *p < 0.05.

Next, to identify OTUs that were affected by DPs, we analysed the differences in the abundance of OTUs between the DP mutants and WT using differential abundance analysis. This revealed consistent differences between WT and the three mutant lines; at the individual sampling dates, a number of bacterial and fungal taxa were consistently enriched in WT roots compared to all three mutant lines (Figures 3A,B and 4A,B). At 17 dpt, this applies to, for example, bacterial genera *Kosakonia*, *Paenibacillus*, *Ochrobacter*, *Xanthomonas, Thermomonas* and *Acidibacter* and fungal genera *Sarcopodium* and *Fuscoporia* (Figure 3A,B). Also, a few microbial taxa including the bacterial genera *Streptomyces*, *Delftia* and the fungal genera *Clonostachys*, *Waitea* and *Coniochaeta* were only enriched in the single mutants.

Microbial genera that were differentially abundant in WT and mutant roots and rhizospheres were completely different at 17 dpt compared to 28 dpt (Figures 3A,B, 4A,B, S5 and S9). At both time points, the number of enriched bacterial and fungal taxa was higher in the rhizosphere of WT than in the single mutants, suggesting that some taxa are attracted to certain DPs (Figures S5 and S6). At 17 dpt, fungal genera including *Botrytis*, *Wallemia*, *Paraphaeospaeria*, *Stemphylium* and *Cystofilobasidium* were consistently enriched in the rhizosphere of WT compared with the mutants. *Staphylococcus* was the bacterial genus that was most enriched in the roots of all the mutants compared to WT, suggesting that DPs restrict its colonisation (Figure 4A). The fungal taxa *Zymoseptoria*, *Dioszegia* and *Itersonilia* were more abundant in WT than in the single mutants (Figure S5B). An overlap of differentially abundant bacterial genera including *Bacteriovorax*, *Pseudoxanthomonas*, *Pedosphaera, Rhodocytophaga* and *Solitalea* was depleted in the rhizospheres of *cps2* and *cps4*.

**FIGURE 5:**
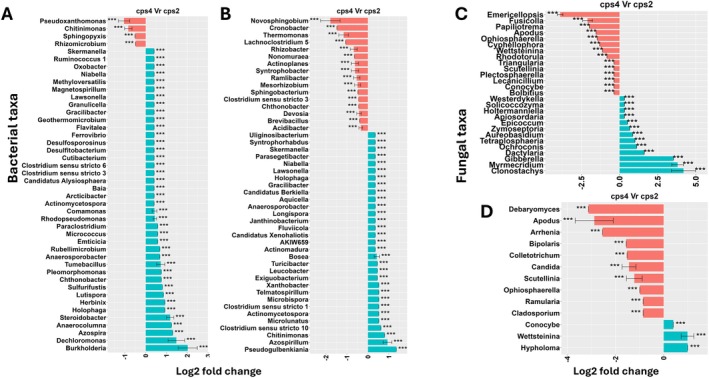
Differentially abundant (log2 fold change) bacterial genera at (A) 17 dpt and (B) 28 dpt and fungal genera at (C) 17 dpt and (D) 28 dpt between roots of rice mutant cps2 and cps4. Data are represented by log fold change (shown as a column), ±SE (shown as error bars) derived from the ANCOM‐BC model. ANCOM‐BC implements Bonferroni correction to adjust for multiple comparisons. The red and green bars represent taxa that are enriched in cps2 and cps4, respectively. The significance of test is indicated as ***p < 0.001, **p < 0.01 and *p < 0.05.

Differential analysis between the single mutants revealed taxa that were significantly enriched in *cps4* relative to the *cps2* mutant in roots at 17 and 28 dpt and in the rhizosphere at 28 dpt (Figures 5 and S7). The differentially abundant taxa were most distinct between root and rhizosphere compartments and between the different time points (Figures 5 and S7). For instance, while *Burkholderia*, *Dechloromonas* and *Azospira* were strongly enriched in *cps4*, *Pseudoxanthomonas*, *Chitinomonas* and *Sphingopyxis* were enriched in *cps2*. Also, bacterial taxa including *Burkholderia*, *Dechloromonas* and *Azospira* were highly enriched in roots of *cps4* at 17 dpt, while *Pseudogulbenkiania*, *Azospirillum* and *Chitinimonas* were enriched at 28 dpt. Likewise, several differentially abundant fungal taxa were detected when comparing *cps2* and *cps4* mutants (Figures 5 and S7).

By comparing each single mutant with the double mutant cps2/4, we identified several differentially abundant taxa (Figures S8–S11). Bacterial taxa *Pseudoxanthomonas* and fungal genera *Emericellopsis*, *Papiliotrema*, *Apodus*, *Cyphellophora* and *Wettsteinina* were consistently enriched in the roots of *cps2* compared to *cps4* and *cps2/4* at 17 dpt, and similarly for taxa such as *Novosphingobium*, *Sphingobacterium* and *Brevibacillus* at 28 dpt (Figures 5 and S8). *Azospira*, *Lutispora*, *Sulfurifustis* and *Pleomorphomonas* were consistently enriched in the roots of *cps4* and *cps2/4* compared to *cps2* at 17 dpt (Figures 5 and S8).

### 3.3 Diterpenoids Affect Associations Between Microorganisms and Nematodes

**FIGURE 6:**
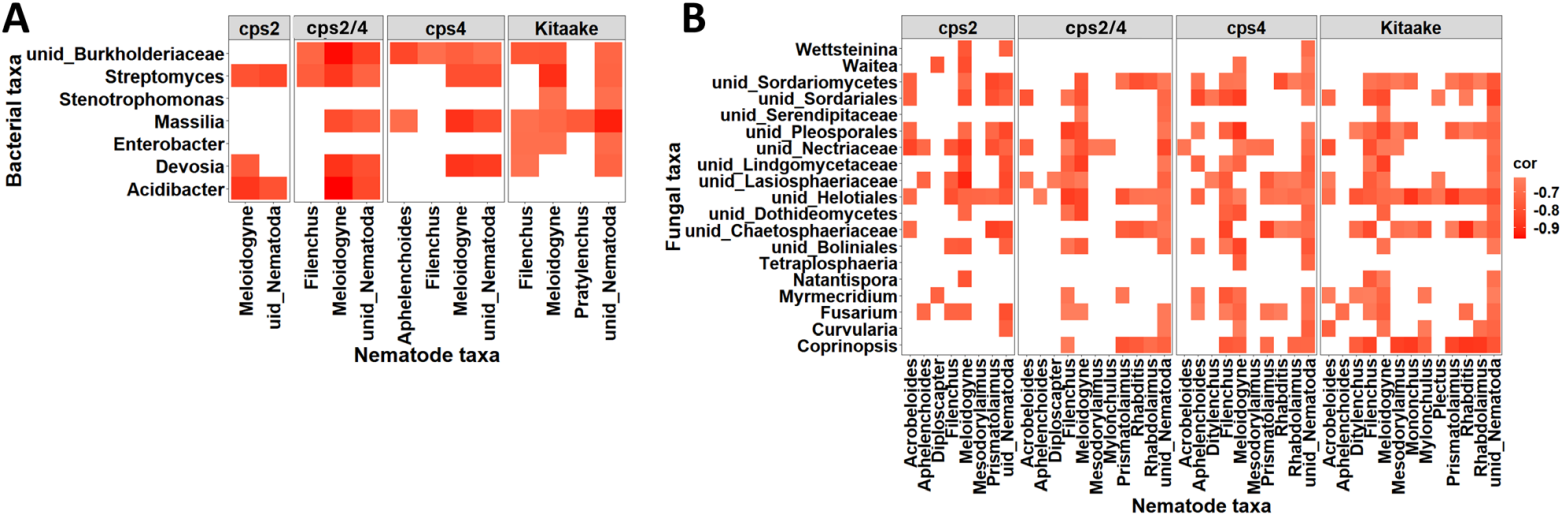
Heat map of microbial‐nematode negative associations in rice wild‐type and mutants. (A) Bacterial‐nematode negative correlations in rice WT and DP impaired mutants. (B) Fungal‐nematode negative correlations in rice WT and DP impaired mutants. Only microorganisms that correlated negatively with *M. graminicola* were used for the visualisation.

Next, we explored putative microbial‐nematode interactions by performing correlation analysis and by examining to what extent patterns of correlations varied between the WT and DP mutants. Overall, patterns of correlations between microbial and nematode taxa were very distinct between the rice genotypes, especially between *cps2* and *cps2/4* (Figure S12). Only a few bacterial taxa including *Acidobacter*, *Devosia*, *Enterobacter*, *Massilia*, *Stenotrophomonas, Streptomyces* and Burkholderiaceae correlated negatively with the parasitic nematode genus *Meloidogyne* (Figure 6A). Of these, only *Streptomyces* correlated negatively with *Meloidogyne* in all genotypes, whereas the other taxa varied between genotypes. Most positive correlations between bacterial taxa and *Meloidogyne* were not affected by DPs (Figure S13A). For instance, the bacterial genera *Sporosarcina*, *Sphingomonas*, *Pseudolabrys*, *Bacillus* and bacterial OTUs assigned to the taxa Methyloligellaceae, Deltaproteobacteria and Acidobacteraceae correlated positively with *Meloidogyne* in most genotypes.

Many fungal taxa correlated negatively with *Meloidogyne* (Figure 6B). These included *Fusarium*, *Coprinopsis*, *Curvularia*, *Myrmecridium*, *Natantispora*, *Tetraplosphaeria*, *Waitea*, *Wettsteinina* and several OTUs belonging to Sordariomycetes. Most of these correlations were observed in WT and *cps4*. In addition, several positive associations were revealed between fungal taxa and *Meloidogyne* in both WT and the mutants (Figure S3B).

## 4 Discussion

### 4.1 Diterpenoids Modulate Root‐Associated Microbiomes in Rice

Previously, we have shown that disruption of rice DP biosynthesis resulted in distinct nematode communities, specifically in the *cps2*, *cps4* and *cps2/4* mutants compared to WT, and most strikingly, these three mutants were more susceptible to *M. graminicola* infection than WT (Desmedt et al. 2022). As these DPs are generally known to be antimicrobial, here we ask if DPs also shape the composition of microbial communities associated with rice roots.

The higher bacterial OTU richness in roots of *cps2* mutants aligns with the higher diversity in microbiomes of *ent*‐CDP derived diterpenoid‐deficient maize (Murphy et al. 2021) and confirms that the antimicrobial activity of these DPs restricts root colonisation and/or proliferation of members of the soil microbiota. The minor effects of mutations on fungal alpha diversity could indicate a selective effect of DPs on a few specific taxa rather than the overall richness. Also, the increased fungal richness in *cps2* roots at 17 dpt confirms both time and distinct DP dependent effects in modulating fungal species.

Disruption of DP synthesis modulated the composition of both bacterial and fungal communities. This corroborates the selective effect that exudation of secondary metabolites imposes on root microbiome assembly shown for other bioactive metabolites such as the benzoxazinoids (Kudjordjie et al. 2019), flavonoids, glucosinolates and camalexin in other plants (Sikder et al. 2022). Together, these results highlight the microbiome modulating potential of plant‐derived defence compounds, offering the prospect to target them in breeding crop varieties with enhanced potential for interacting positively with root microbiota.

We found that the relative abundances of bacterial phyla and fungal classes varied between genotypes and sampling times. At 28 dpt, only *cps4* exhibited any significant difference in rhizosphere communities. At the OTU level, a similar pattern emerged, where the compositional differences between WT and the mutants were more pronounced in the roots than in the rhizosphere and at the earlier (17 dpt) than later (28 dpt) sampling time. These results align with a study which showed that a vast array of triterpenes influenced the assembly of *Arabidopsis* root‐associated microbiota (Huang et al. 2019). Moreover, differences in relative abundances of bacterial taxa in roots between single mutants at 17 dpt indicate distinct effects of *ent*‐CDP derived (*OsCPS2*‐dependent) versus *syn*‐CDP derived (*OsCPS4*‐dependent) DPs in rice microbiome assembly. For instance, the strong enrichment of Actinobacteria in roots of *cps2*, and the higher species richness in both root and rhizosphere of *cps2* than *cps4* suggests that *ent*‐CDP derived DPs exert a stronger selection effect on rice bacterial community assembly than *syn*‐CDP derived DPs.

As noted, the differences in microbial communities were more evident at the early sampling than at the later sampling. This suggests an early structuring effect of DPs on rice‐associated microbiomes, which may be caused by high DP secretion in younger plants. Plants are known to produce high quantities of defensive metabolites at early developmental stages, to ensure protection of seedlings (Kudjordjie et al. 2019), and young rice seedlings are known also to exude DPs (Toyomasu et al. 2008).

### 4.2 Diterpenoids Affect Putative Microbe‐Nematode Interactions

Previous work has shown that co‐variation in plant production of secondary metabolites and susceptibility to root‐knot nematodes may be modulated via microbial responses (Sikder et al. 2022). Therefore, we wanted to further explore whether the DP‐induced changes in nematode communities, most notably the enrichment of *M. graminicola* in the DP mutants (Desmedt et al. 2022), were related to microbial changes between WT and the mutants. Comparing correlations between nematode and microbial taxa across the four rice genotypes, it is apparent that overall patterns of correlations between nematode taxa and individual bacterial and fungal OTUs are distinct. This clearly suggests that DPs play a role in tritrophic inter‐kingdom interactions within the root‐associated biota. Only a limited number of bacterial taxa correlated with *Meloidogyne*, notably *Streptomyces*, which correlated negatively with *Meloidogyne* in all rice genotypes. *Stenotrophomonas* and *Enterobacter* only correlated (negatively) with *Meloidogyne* in WT, and *Massilia* as well as an OTU assigned to Burkholderiaceae correlated negatively with *Meloidogyne* in all genotypes except *cps2*, whereas *Acidibacter* correlated negatively with *Meloidogyne* in the two mutant lines carrying the *cps2* mutation (*cps2* and *cps2/4*). The negative correlations suggest that these bacterial taxa play a role in suppressing *M. graminicola* infectivity and corroborate previous reports on nematode‐suppressive activity exerted by strains within these taxa. For instance, *Enterobacter* strains (Duponnois et al. 1999; Oliveira et al. 2007; Oh et al. 2018; Zhao et al. 2022), *Stenotrophomonas* strains (Mekete et al. 2009; Groover et al. 2020), *Burkholderia* species (Zhang et al. 2022; Kim et al. 2023) and many *Streptomyces* species (Nimnoi and Ruanpanun 2020; Topalović et al. 2020; Hu et al. 2022) are known to be nematicidal. In addition, the chitinolytic activity of *Massilia* has been suggested to be involved in the suppression of plant parasitic nematodes (Cretoiu et al. 2013). In a previous study, we likewise reported negative correlations between *Acidobacter* and *M. incognita* in *Arabidopsis* roots (Sikder et al. 2021).

### 4.3 Diterpenoids Affect the Abundance of Specific Bacterial and Fungal Taxa

Having established that the DP mutants and WT assembled distinct root microbiomes, we aimed to identify bacterial and fungal taxa that were differentially represented in mutant and WT microbiomes. In line with the higher bacterial OTU richness in the mutants, a considerable number of taxa were enriched in the microbiomes of these DP‐deficient lines, whereas only a limited number of taxa had a higher relative abundance in WT. In general, the comparison between WT and the individual mutants revealed that a few taxa were consistently enriched either in WT or the mutants. This consistent pattern suggests that DPs exert an antimicrobial effect on specific microbial taxa during the establishment of the root microbiome.

Bacterial taxa that correlated negatively with *M. graminicola* , for example, *Enterobacter*, *Xanthomonas*, *Sphingobacterium* and *Paenibacillus*, were strongly enriched in the roots of WT relative to the DP mutants at the early growth stages of rice development. This could suggest that the higher resistance in WT than in the mutants could partly be attributed to the higher abundances of specific putative *M. graminicola* ‐antagonistic microbial taxa in the WT. Furthermore, specific microbial taxa, for example, the bacterial genera *Streptomyces*, *Delftia*, and the fungal genera *Clonostachys*, *Waitea* and *Coniochaeta*, were consistently enriched in the roots of single mutants but not in the double mutant at 17 dpt. Conversely, several microbial taxa were enriched in the roots and rhizosphere of the double mutant at both 17 dpt and 28 dpt. We speculate that in the double mutant, the disruption of both *OsCPS2* (i.e., DPs derived from *ent*‐CDP) and *OsCPS4* (i.e., DPs derived from *syn*‐CDP) genes differentially affects individual microbial taxa.

The higher number of differentially abundant bacterial genera identified in roots of *cps4* at 17 dpt and the enrichment of specific fungal taxa in *cps2* roots at 28 dpt, further support the distinct effects of DPs derived from *ent*‐CDP and *syn*‐CDP branches of the rice DP metabolic network. Lu et al. (2018) reported that *OsCPS2*‐dependent DPs were active in rice plant defence against both the fungal pathogen *Magnaporthe oryzae* and bacterial pathogen *X. oryzae* , while *OsCPS4*‐dependent DPs were involved in non‐host resistance against the turfgrass fungal pathogen *Magnaporthe poae*. The DPs dependent on *OsCPS2* are mainly the phytocassanes and abietoryzins, while the *OsCPS4*‐dependent DPs include the momilactones and oryzalexin S (Toyomasu et al. 2014). The activity of the *ent*‐CDP and *syn*‐CDP derived DPs against specific fungal species have been widely reported (reviewed in (Valletta et al. 2023)). Previous studies reported that momilactones did not inhibit the rice blast fungi *M. oryzae* or *Fusarium fujikuroi*, the causative agent of the bakanae ‘foolish seedling’ disease (Xu et al. 2012). Also, studies have shown that momilactone B is more active against *Botrytis cinerea*, *Fusarium solani* and *Colletrotrichum gloeosporioides* than momilactone A, whereas neither of these momilactones affect *Fusarium oxysporum* (Fukuta et al. 2007). Moreover, the consistent enrichment of specific microbial taxa in the mutants reported here suggests that the depletion of specific DPs enables their establishment in the root (Zarraonaindia et al. 2015). Altogether, the present findings demonstrate that DPs have a profound effect on individual microbial taxa, exhibiting some specificity in their interactions, and overall modulating the assembly of rice‐associated microbiomes.

## 5 Conclusion

Our study provided detailed evidence for the various roles of DPs in structuring the root‐associated microbiome of rice plants and showed indications of microbiome‐mediated effects on the parasitic root‐knot nematode genus *Meloidogyne*. We found that disruption of the two branches (*cps2* and *cps4*) of the rice DP biosynthetic network affected the assembly of rice root microbiomes, with distinct effects on specific microbial taxa. Moreover, DP mutations caused significant shifts in bacterial diversity. Bacterial OTU richness was higher in the *cps2* and *cps2/4* mutants compared to WT in the roots at both sampling times and in the rhizosphere at 28 dpt. It appears that DPs have a ‘gatekeeper’ role; that is, DPs block certain microbial taxa from establishing themselves in the rice root‐associated microbiome. Interestingly, several bacterial and fungal taxa that correlated negatively with *M. graminicola* or are known to control root‐knot nematodes were enriched in WT compared to the mutants. This is interesting, as *M. graminicola* was previously shown to be less abundant in the WT than in the mutant lines. Bacterial taxa that correlated negatively with *M. graminicola* , for example, *Enterobacter*, *Xanthomonas*, *Sphingobacterium* and *Paenibacillus*, were strongly enriched in the roots of WT relative to the DP mutants at the early growth stages of rice development. These taxa, together with the highly enriched genus *Kosakonia*, are antagonists of microbial pathogens as well. These findings provide insights into how rice‐secreted DPs mediate the assembly of root microbiomes, including the recruitment of beneficial microbes suppressing plant parasitic nematodes.

The higher resistance of the WT and facilitation of potential microbial taxa with nematicidal activity could be exploited via breeding programs targeting the development of rice varieties with DP profiles conferring enhanced resistance against plant parasitic nematodes. However, breeding of new varieties could be impeded by our limited understanding of the mechanisms of DP‐mediated effects in the assembly of host‐associated microbiomes. Future studies focusing on deciphering the mechanisms of complex interactions are needed to facilitate the breeding of more resistant cultivars.

## Author Contributions

**Enoch Narh Kudjordjie:** methodology, software, data curation, investigation, validation, formal analysis, visualization, writing – original draft, writing – review and editing, conceptualization, resources. **Willem Desmedt:** conceptualization, methodology, data curation, investigation, validation, writing – review and editing, resources. **Tina Kyndt:** conceptualization, methodology, validation, supervision, funding acquisition, project administration, writing – review and editing, resources. **Mogens Nicolaisen:** conceptualization, methodology, validation, supervision, resources, project administration, writing – review and editing, funding acquisition. **Reuben J. Peters:** conceptualization, methodology, validation, supervision, funding acquisition, project administration, resources, writing – review and editing. **Mette Vestergård:** conceptualization, methodology, validation, supervision, funding acquisition, project administration, resources, writing – original draft, writing – review and editing.

## Conflicts of Interest

The authors declare no conflicts of interest.

## Supporting information


**Figure S1.** (A) bacterial and (B) fungal sequence reads and OTUs in samples used in this study. Rarefaction curves showing the coverage of (C) bacterial and (D) fungal OTU richness (species richness in number of OTUs) as a function of sequencing depth (sample size in number of reads).
**Figure S2.** Bacterial Shannon diversity in root and rhizosphere of WT and mutant rice at (A) 17 and (B) 28 dpt. Fungal alpha diversity (Observed and Shannon) in root and rhizosphere of WT and mutant rice at (C) 17 and (D) 28 dpt.
**Figure S3.** Microbial community composition in soil and rice lines. Principal Coordinates Analysis (PCoA) of rice WT and mutants (A) bacterial and (B) fungal communities. Analysis was performed using datasets from both 17 and 28 dpt.
**Figure S4.** Principal Coordinates Analysis of microbial communities in rice WT and mutants using Bray‐Curtis dissimilarity distances. PCoA plots of bacterial community in root and rhizosphere at (A) 17 and (B) 28 dpt. PCoA plots of fungal community in root and rhizosphere at (C) 17 and (D) 28 dpt.
**Figure S5.** Differentially abundant (log2 fold change) microbial genera in the rhizosphere of Kitaake (wild‐type rice) and mutants. Differentially abundant (A) bacterial and (B) fungal genera between Kitaake (red) and individual mutants (green) at 17 dpt. Data are represented by log fold change (shown as a column), ±SE (shown as error bars) derived from the ANCOM‐BC model. The significance of test is indicated as ***p < 0.001, **p < 0.01 and *p < 0.05.
**Figure S6.** Differentially abundant (log2 fold change) microbial genera in the rhizosphere of Kitaake (wild‐type rice) and mutants. Differentially abundant (A) bacterial and (B) fungal genera between Kitaake (red) and individual mutants (green) at 28 dpt. Data are represented by log fold change (shown as a column), ±SE (shown as error bars) derived from the ANCOM‐BC model. The significance of test is indicated as ***p < 0.001, **p < 0.01 and *p < 0.05.
**Figure S7.** Differentially abundant (log2 fold change) microbial genera in the rhizosphere of cps2 (red) and cps4 (green). Differentially abundant (A) bacterial and (B) fungal genera between Kitaake and individual mutants at 28 dpt. Data are represented by log fold change (shown as a column), ±SE (shown as error bars) derived from the ANCOM‐BC model. The significance of test is indicated as ***p < 0.001, **p < 0.01 and *p < 0.05.
**Figure S8.** Differentially abundant (log2 fold change) root bacterial taxa between cps2 (red) and cps2/4 (green) at (A) 17 dpt and (B) 28 dpt and fungal genera at (C) 17 dpt and (D) 28 dpt. Data are represented by log fold change (shown as a column), ±SE (shown as error bars) derived from the ANCOM‐BC model. The significance of test is indicated as ***p < 0.001, **p < 0.01 and *p < 0.05.
**Figure S9.** Differentially abundant (log2 fold change) root bacterial taxa between cps4 (red) and cps2/4 (green) at (A) 17 dpt and (B) 28 dpt and fungal genera at (C) 17 dpt and (D) 28 dpt. Data are represented by log fold change (shown as a column), ±SE (shown as error bars) derived from the ANCOM‐BC model. The significance of test is indicated as ***p < 0.001, **p < 0.01 and *p < 0.05.
**Figure S10.** Differentially abundant (log2 fold change) rhizosphere bacterial taxa between cps2 (red) and cps2/4 (green) at (A) 17 dpt and (B) 28 dpt and fungal genera at (C) 17 dpt and (D) 28 dpt. Data are represented by log fold change (shown as a column), ±SE (shown as error bars) derived from the ANCOM‐BC model. The significance of test is indicated as ***p < 0.001, **p < 0.01 and *p < 0.05.
**Figure S11.** Differentially abundant (log2 fold change) rhizosphere bacterial taxa between cps4 (red) and cps2/4 (green) at (A) 17 dpt and (B) 28 dpt and fungal genera at (C) 17 dpt and (D) 28 dpt. Data are represented by log fold change (shown as a column), ±SE (shown as error bars) derived from the ANCOM‐BC model. The significance of test is indicated as ***p < 0.001, **p < 0.01 and *p < 0.05.
**Figure S12.** Heat map of microbial‐nematode associations in rice WT and mutants. Both microbes that correlated positively and negatively with *M. graminicola* were used for the visualisation.
**Figure S13.** Heat map of microbial‐nematode associations in rice WT and mutants. Only microbes that positively correlated with *M. graminicola* were used for the visualisation.
**Table S1.** Primer sets used in this study for the amplification of bacterial 16S rRNA V5‐7 genes. Multiplex identifiers (MID) are indicated in bold in forward primer (799F).
**Table S2.** Mean, median and range of reads per compartment for fungal and bacterial libraries at different days post inoculation (DPI).
**Table S3.** Permutation analysis of variance (PERMANOVA) using ‘adonis’ test on Bray‐Curtis distance matrices for bacterial and fungal community dissimilarity assessment using 1000 permutations.
**Table S4.** PERMANOVA (pairwise‐adonis) between rice mutants cps2 and cps4.

## Data Availability

The raw bacterial and fungal sequence data has been uploaded to Sequence Read Archive (SRA) bioproject number PRJNA1101406 with accession numbers (SUB14384063 and SUB14384196), respectively. The raw nematode sequence data can be assessed using the bioproject number SRA bioproject number PRJNA797929 (Desmedt et al. 2022). Supporting Information are available in the figshare repository, https://doi.org/10.6084/m9.figshare.28540697.
