# Supplementary material for: Diterpenoid Phytoalexins Shape Rice Root Microbiomes and Their Associations With Root Parasitic Nematodes
Source: Environ Microbiol. 2025 Mar 28;27(4):e70084. doi: 10.1111/1462-2920.70084 (PMC11950907; doi:10.1111/1462-2920.70084)
Supplement: Supplementary file 1 — Figure S1. (A) bacterial and (B) fungal sequence reads and OTUs in samples used in this study. Rarefaction curves showing the coverage of (C) bacterial and (D) fungal OTU richness (species richness in number of OTUs) as a function of sequencing depth (sample size in number of reads). Figure S2. Bacterial Shannon diversity in root and rhizosphere of WT and mutant rice at (A) 17 and (B) 28 dpt. Fungal alpha diversity (Observed and Shannon) in root and rhizosphere of WT and mutant rice at (C) 17 and (D) 28 dpt. Figure S3. Microbial community composition in soil and rice lines. Principal Coordinates Analysis (PCoA) of rice WT and mutants (A) bacterial and (B) fungal communities. Analysis was performed using datasets from both 17 and 28 dpt. Figure S4. Principal Coordinates Analysis of microbial communities in rice WT and mutants using Bray‐Curtis dissimilarity distances. PCoA plots of bacterial community in root and rhizosphere at (A) 17 and (B) 28 dpt. PCoA plots of fungal community in root and rhizosphere at (C) 17 and (D) 28 dpt. Figure S5. Differentially abundant (log2 fold change) microbial genera in the rhizosphere of Kitaake (wild‐type rice) and mutants. Differentially abundant (A) bacterial and (B) fungal genera between Kitaake (red) and individual mutants (green) at 17 dpt. Data are represented by log fold change (shown as a column), ±SE (shown as error bars) derived from the ANCOM‐BC model. The significance of test is indicated as ***p < 0.001, **p < 0.01 and *p < 0.05. Figure S6. Differentially abundant (log2 fold change) microbial genera in the rhizosphere of Kitaake (wild‐type rice) and mutants. Differentially abundant (A) bacterial and (B) fungal genera between Kitaake (red) and individual mutants (green) at 28 dpt. Data are represented by log fold change (shown as a column), ±SE (shown as error bars) derived from the ANCOM‐BC model. The significance of test is indicated as ***p < 0.001, **p < 0.01 and *p < 0.05. Figure S7. Differentially abundant (log2 fo [file EMI-27-e70084-s001.pdf]

Supplementary files

Phytoalexin diterpenoids shape rice root microbiomes and their associations with root parasitic nematodes

Enoch Narh Kudjordjie[1], Willem Desmedt[2,3,4], Tina Kyndt[2], Mogens Nicolaisen[1], Reuben J. Peters[5*], Mette Vestergård[1*]

[1]Department of Agroecology, Faculty of Technical Sciences, Aarhus University, Slagelse 4200, Denmark.

[2]VIB Center for Plant Systems Biology, Ghent 9052, Belgium.

[3]Research Group Epigenetics and Defence, Department of Biotechnology, Ghent University, Ghent 9000, Belgium; [4]Department of Plant Biotechnology and Bioinformatics, Ghent University, Ghent 9052, Belgium.

[5]Roy J. Carver Department of Biochemistry, Biophysics & Molecular Biology, Iowa State University, Ames, IA 50011, USA.

*Correspondence: Reuben J. Peters (rjpeters@iastate.edu); Mette Vestergård (mvestergard@agro.au.dk)

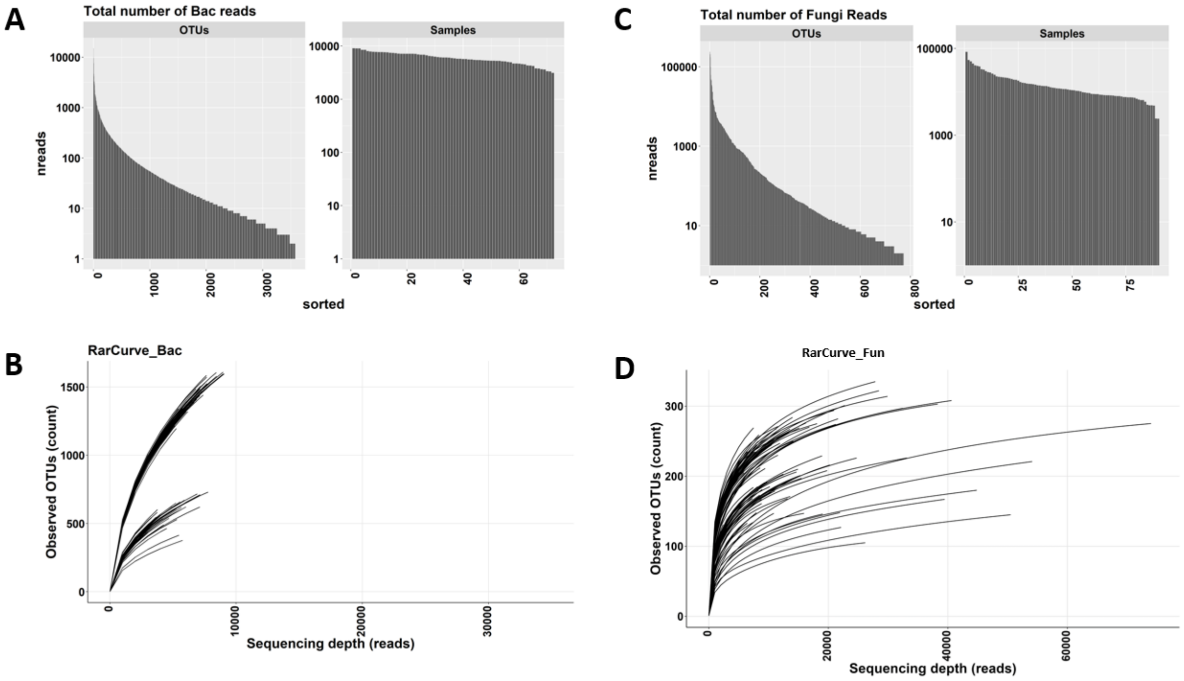

Fig. S1. (**A**) bacterial and (**B**) fungal sequence reads and OTUs in samples used in this study. Rarefaction curves showing the coverage of (**C**) bacterial and (**D**) fungal OTU richness (species richness in number of OTUs) as a function of sequencing depth (sample size in number of reads).

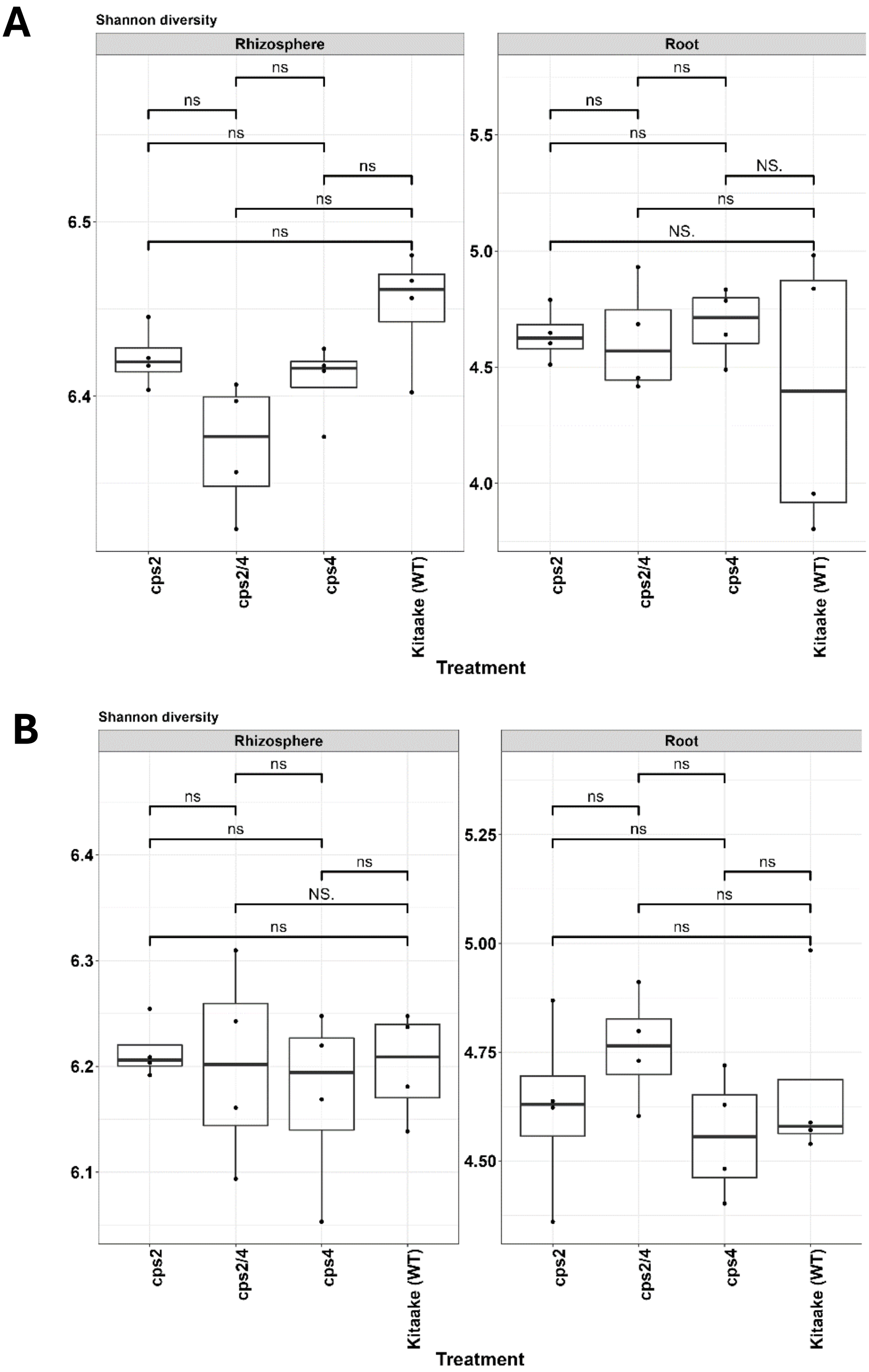

Fig. S2. Bacterial Shannon diversity in root and rhizosphere of WT and mutant rice at **A**) 17 and **B**) 28 dpt.

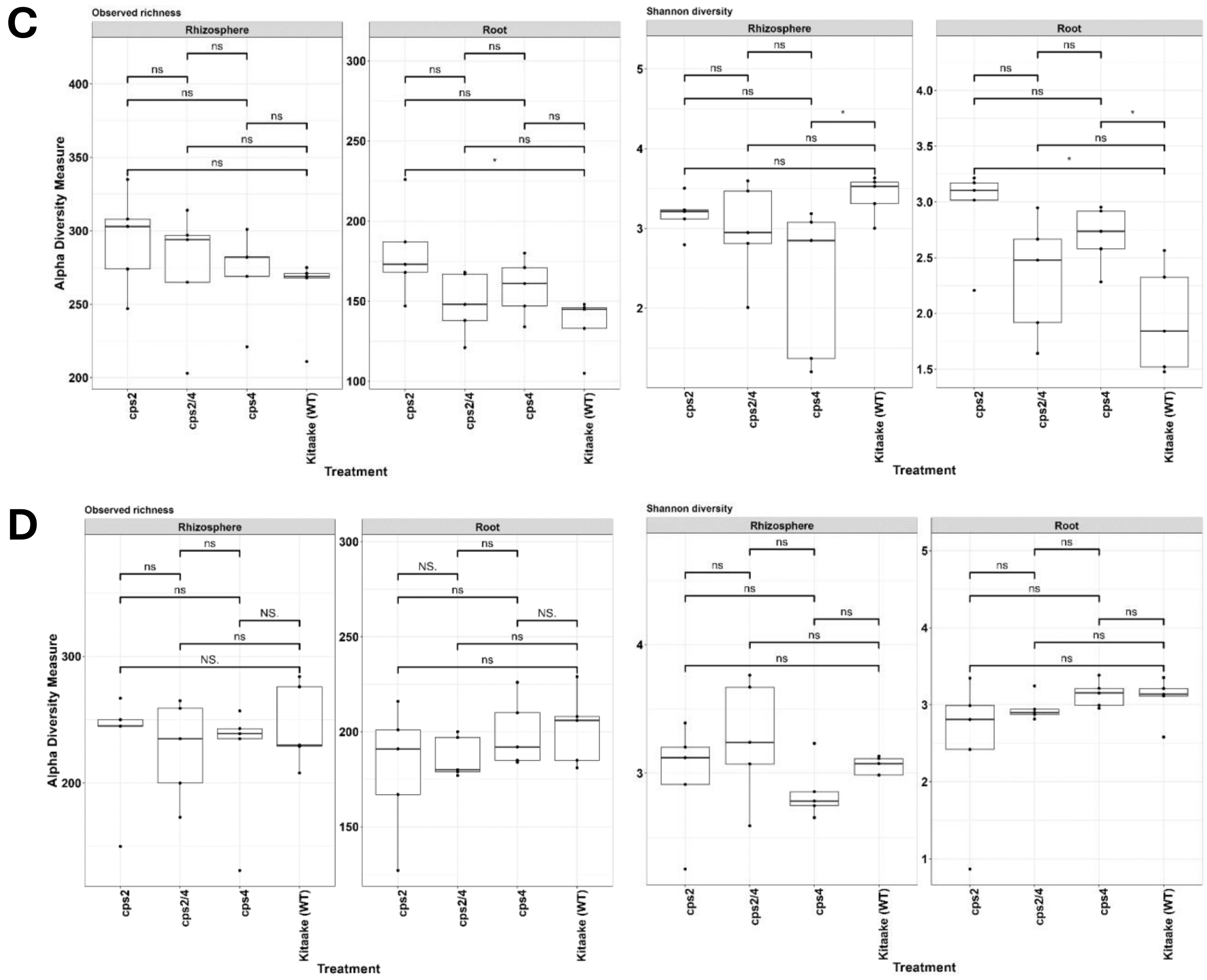

Fig. S2. Fungal alpha diversity (Observed and Shannon) in root and rhizosphere of WT and mutant rice at **C**) 17 and **D**) 28 dpt.

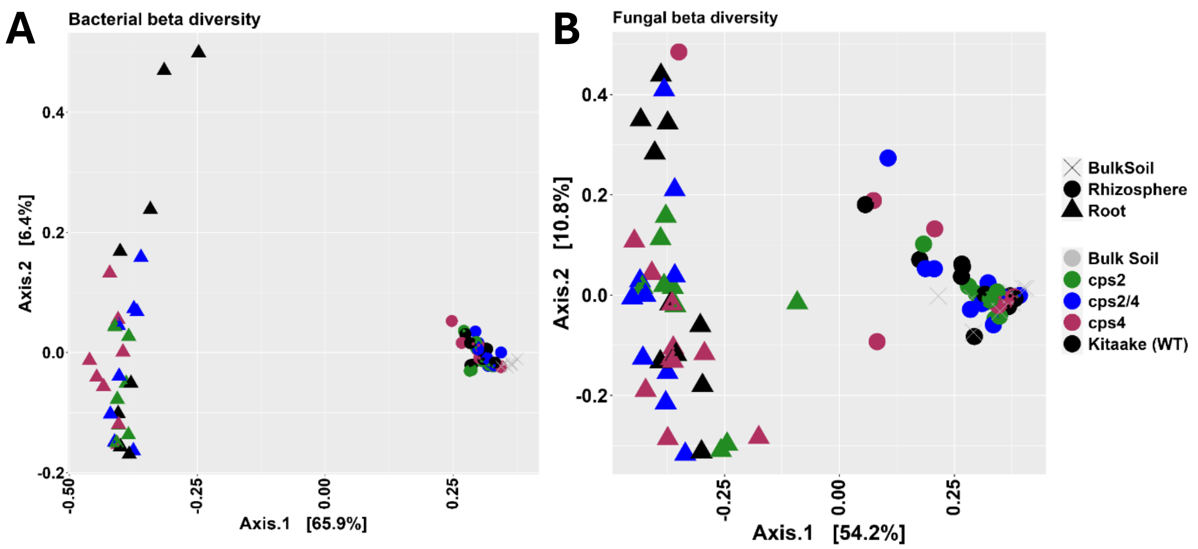

Fig. S3. Microbial community composition in soil and rice lines. Principal coordinates analysis (PCoA) of rice WT and mutants **A**) bacterial community and **B**) fungal communities. Analysis was performed using datasets from both 17 and 28 dpt.

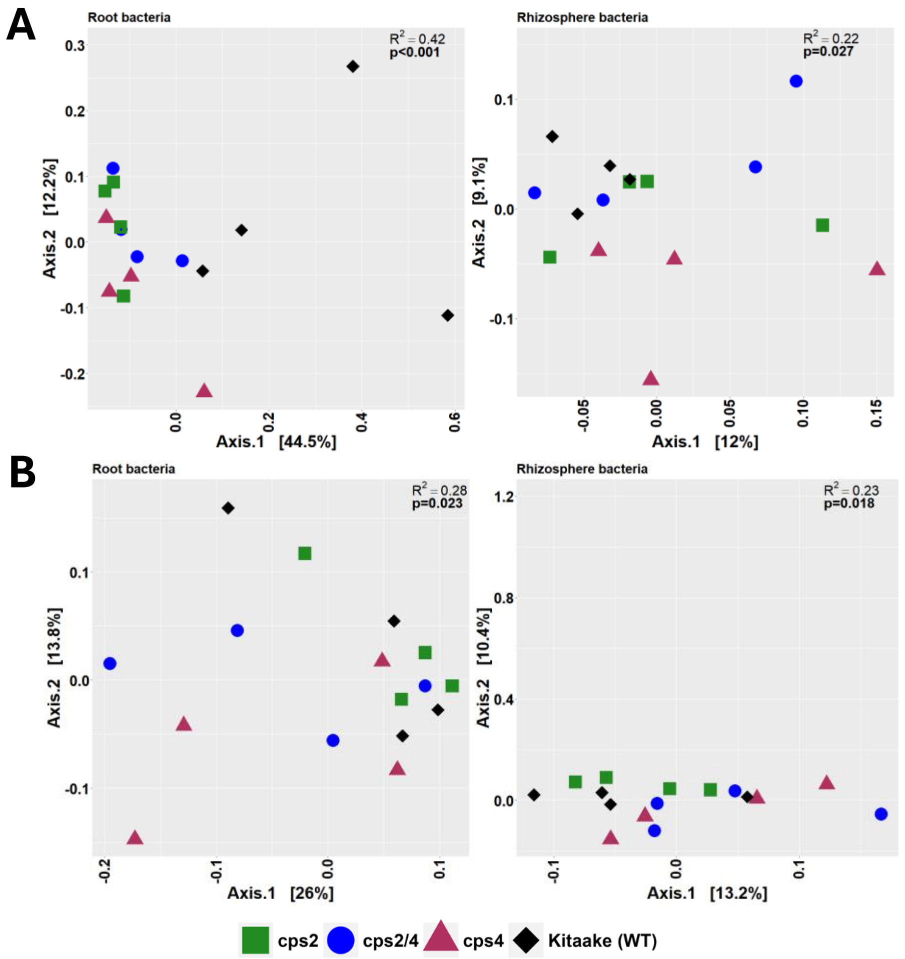

Fig. S4. Principal Coordinates Analysis of microbial communities in rice WT and mutants using Bray-Curtis dissimilarity distances. PCoA plots of bacterial community in root and rhizosphere at **A**) 17 and **B**) 28 dpt.

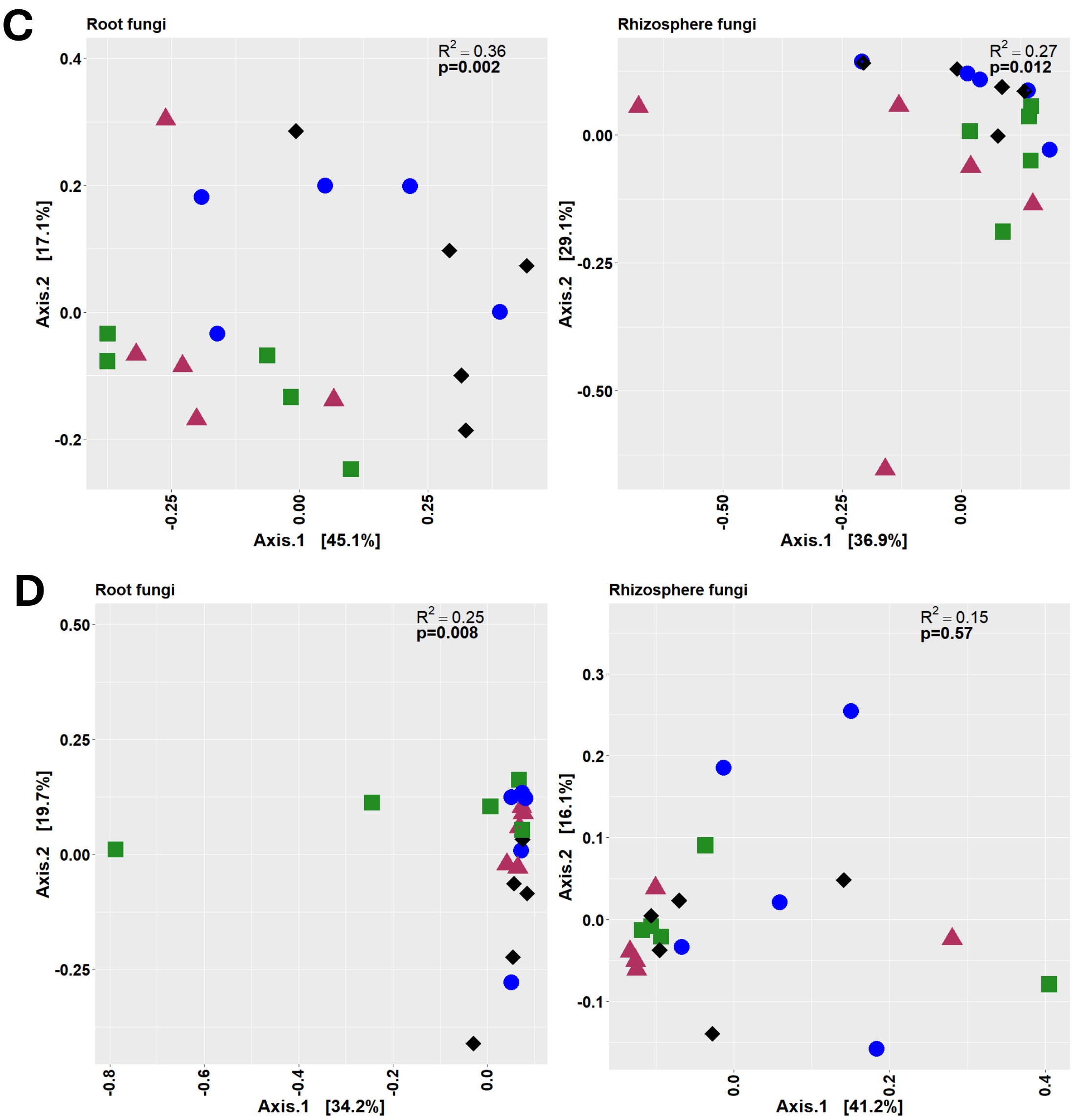

Fig. S4. Principal Coordinates Analysis of microbial communities in rice WT and mutants using Bray-Curtis dissimilarity distances. PCoA plots of fungal community in root and rhizosphere at **C**) 17 and **D**) 28 dpt.

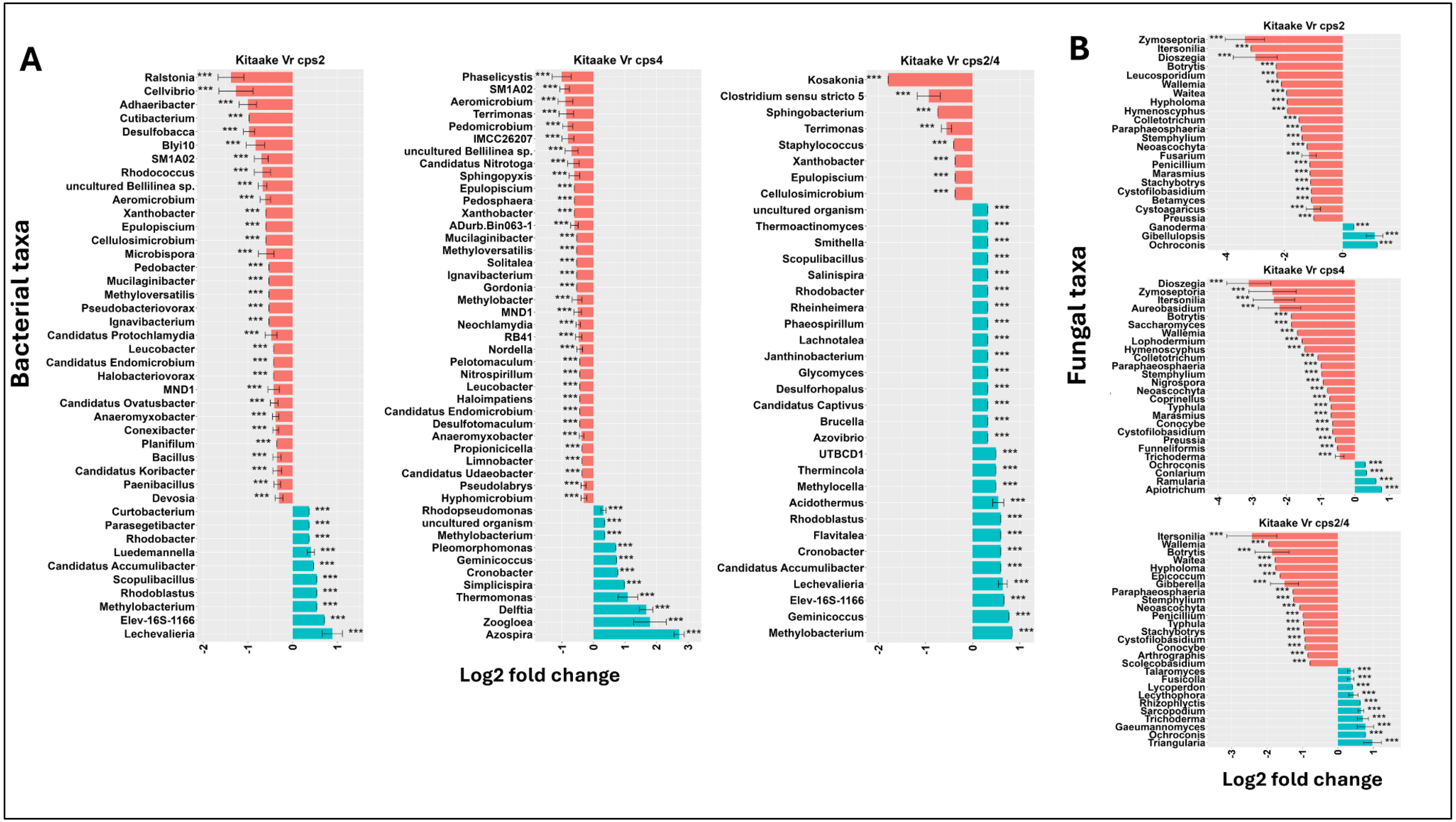

Fig. S5. Differentially abundant (Log2 fold change) microbial genera in the rhizosphere of Kitaake (wildtype rice) and mutants. Differentially abundant A) bacterial and B) fungal genera between Kitaake (red) and individual mutants (green) at 17 dpt. Data are represented by log fold change (shown as a column), ±SE (shown as error bars) derived from the ANCOM-BC model. The significance of test is indicated as ***for $p < 0.001$, ** for $p < 0.01$ and * for $p < 0.05$.

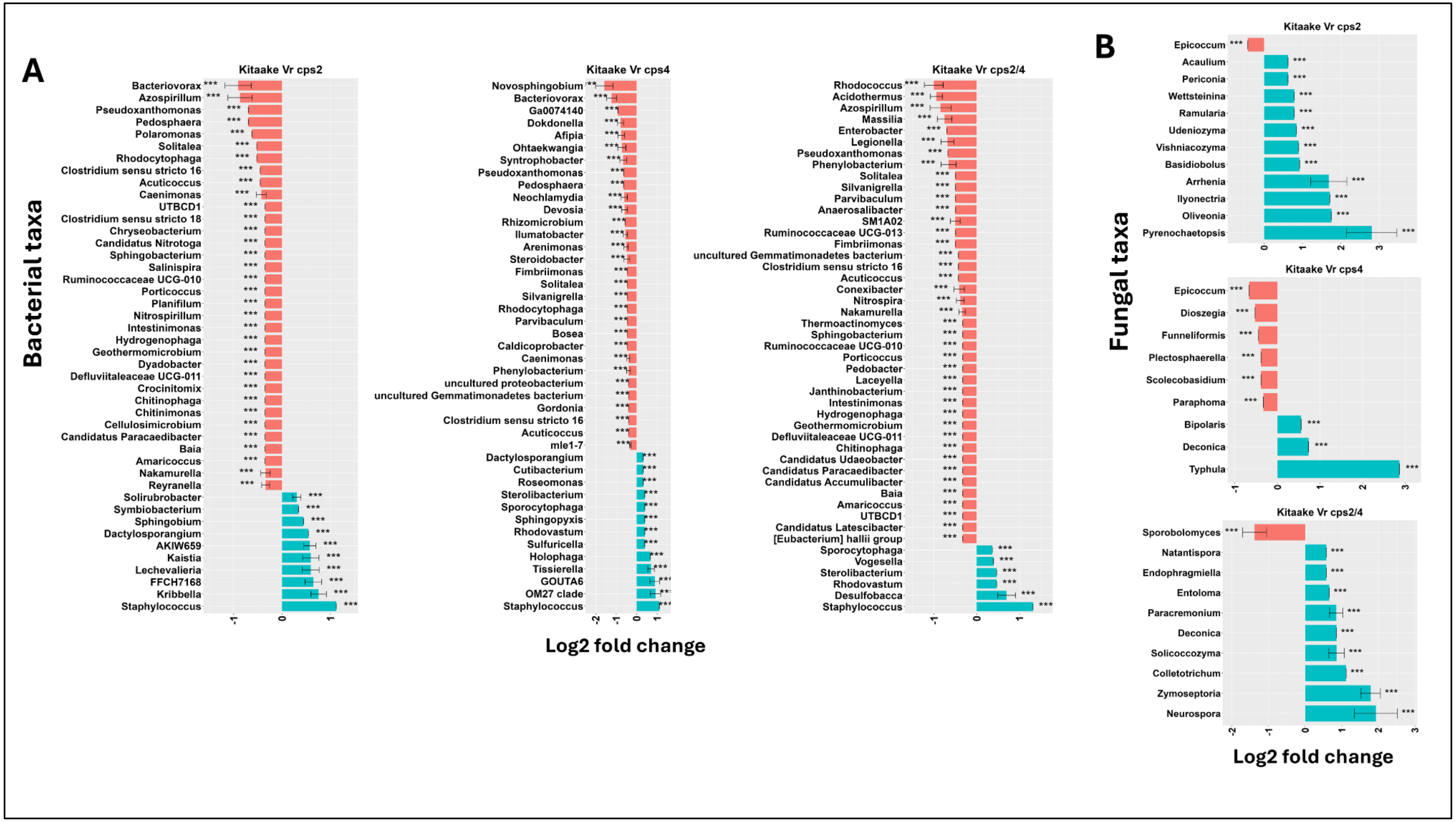

Fig. S6. Differentially abundant (Log2 fold change) microbial genera in the rhizosphere of Kitaake (wildtype rice) and mutants. Differentially abundant A) bacterial and B) fungal genera between Kitaake (red) and individual mutants (green) at 28 dpt. Data are represented by log fold change (shown as a column), ±SE (shown as error bars) derived from the ANCOM-BC model. The significance of test is indicated as ***for $p < 0.001$, ** for $p < 0.01$ and * for $p < 0.05$.

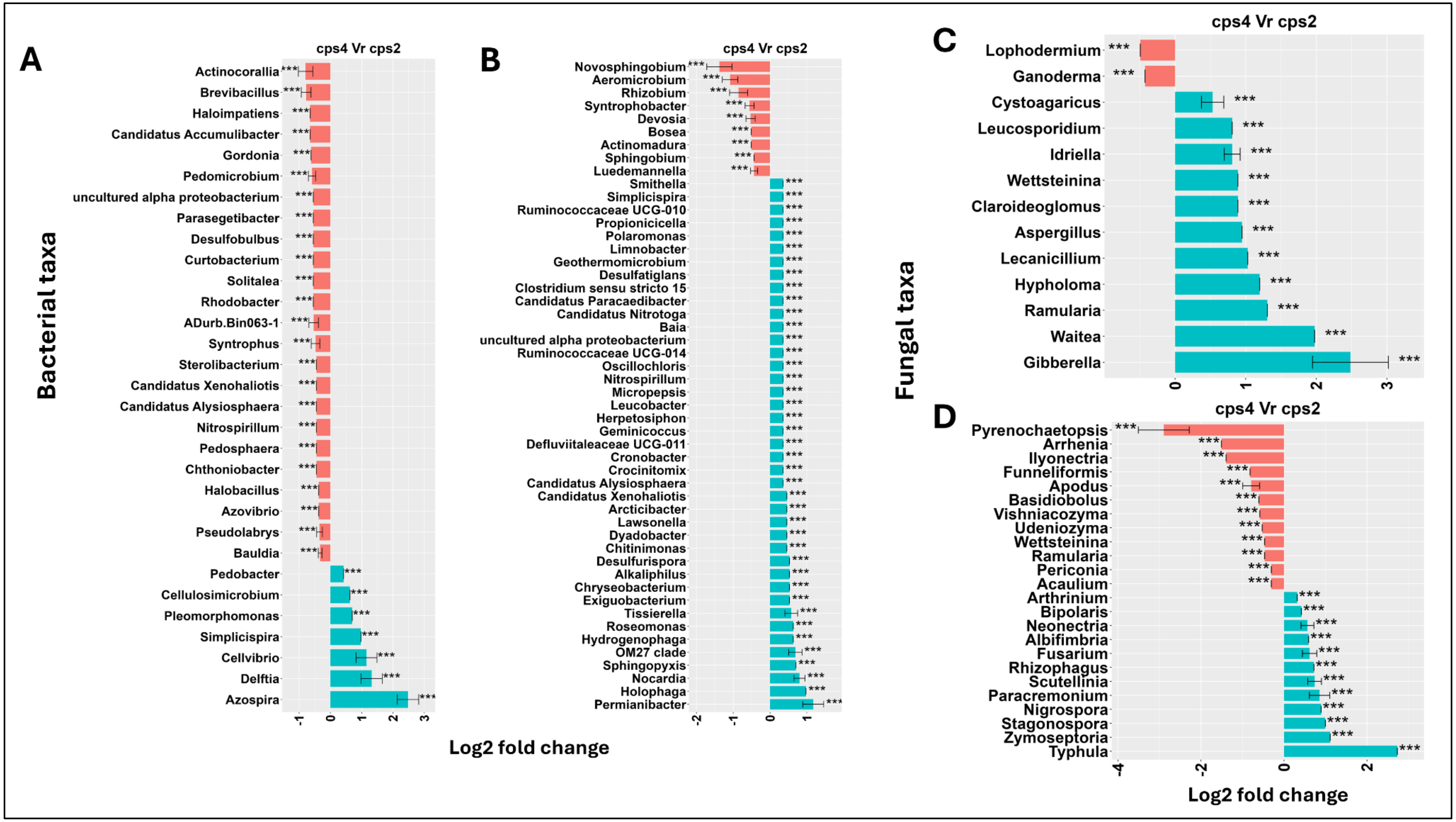

Fig. S7. Differentially abundant (Log2 fold change) microbial genera in the rhizosphere of cps2 (red) and cps4 (green). Differentially abundant A) bacterial and B) fungal genera between Kitaake and individual mutants at 28 dpt. Data are represented by log fold change (shown as a column), ±SE (shown as error bars) derived from the ANCOM-BC model. The significance of test is indicated as ***for $p < 0.001$, ** for $p < 0.01$ and * for $p < 0.05$.

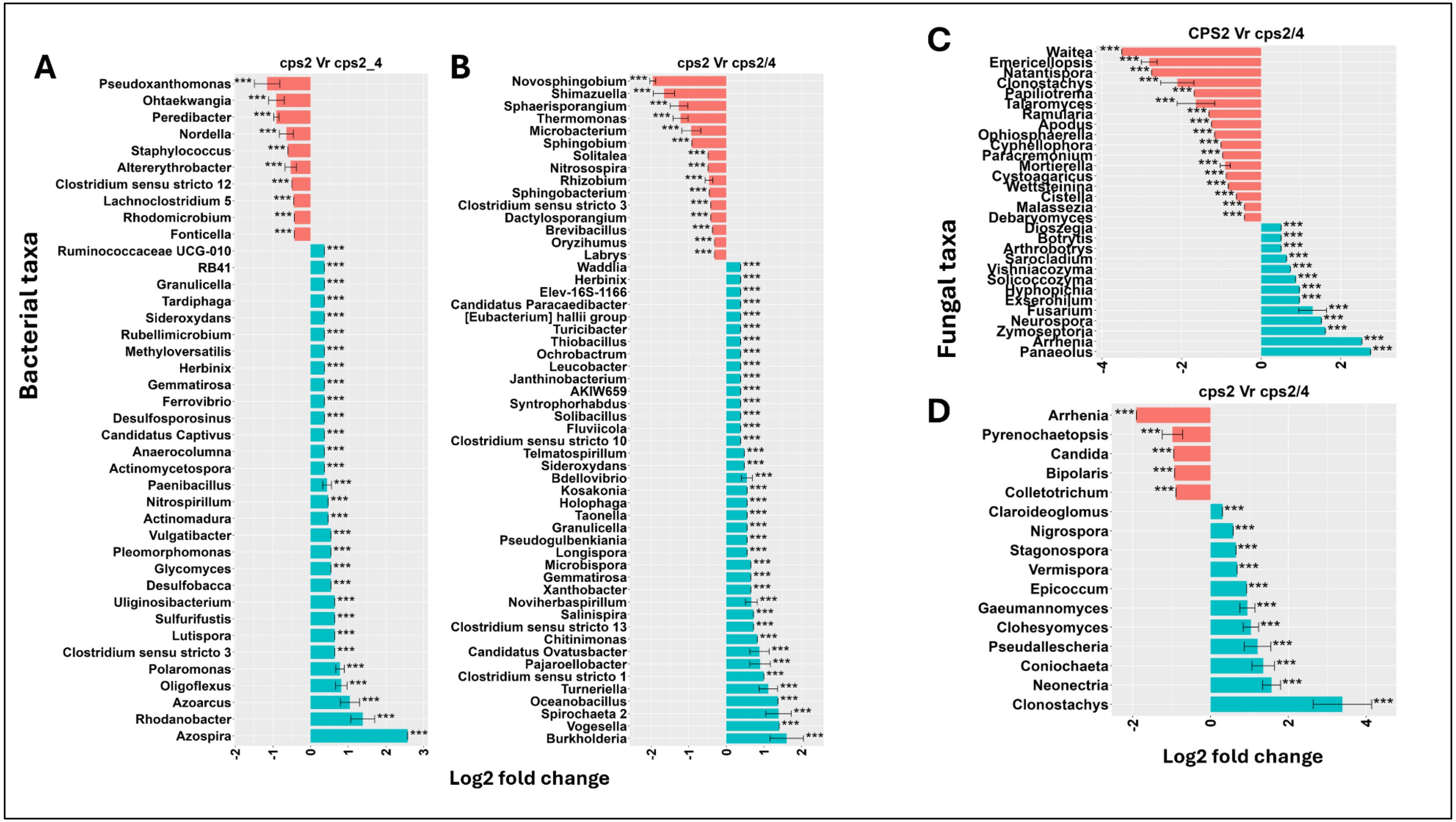

Fig. S8. Differentially abundant (Log2 fold change) root bacterial taxa between cps2 (red) and cps2/4 (green) at A)17dpt and B) 28dpt and fungal genera at C) 17dpt and D) 28dpt. Data are represented by log fold change (shown as a column), ±SE (shown as error bars) derived from the ANCOM-BC model. The significance of test is indicated as ***for $p < 0.001$, ** for $p < 0.01$ and * for $p < 0.05$.

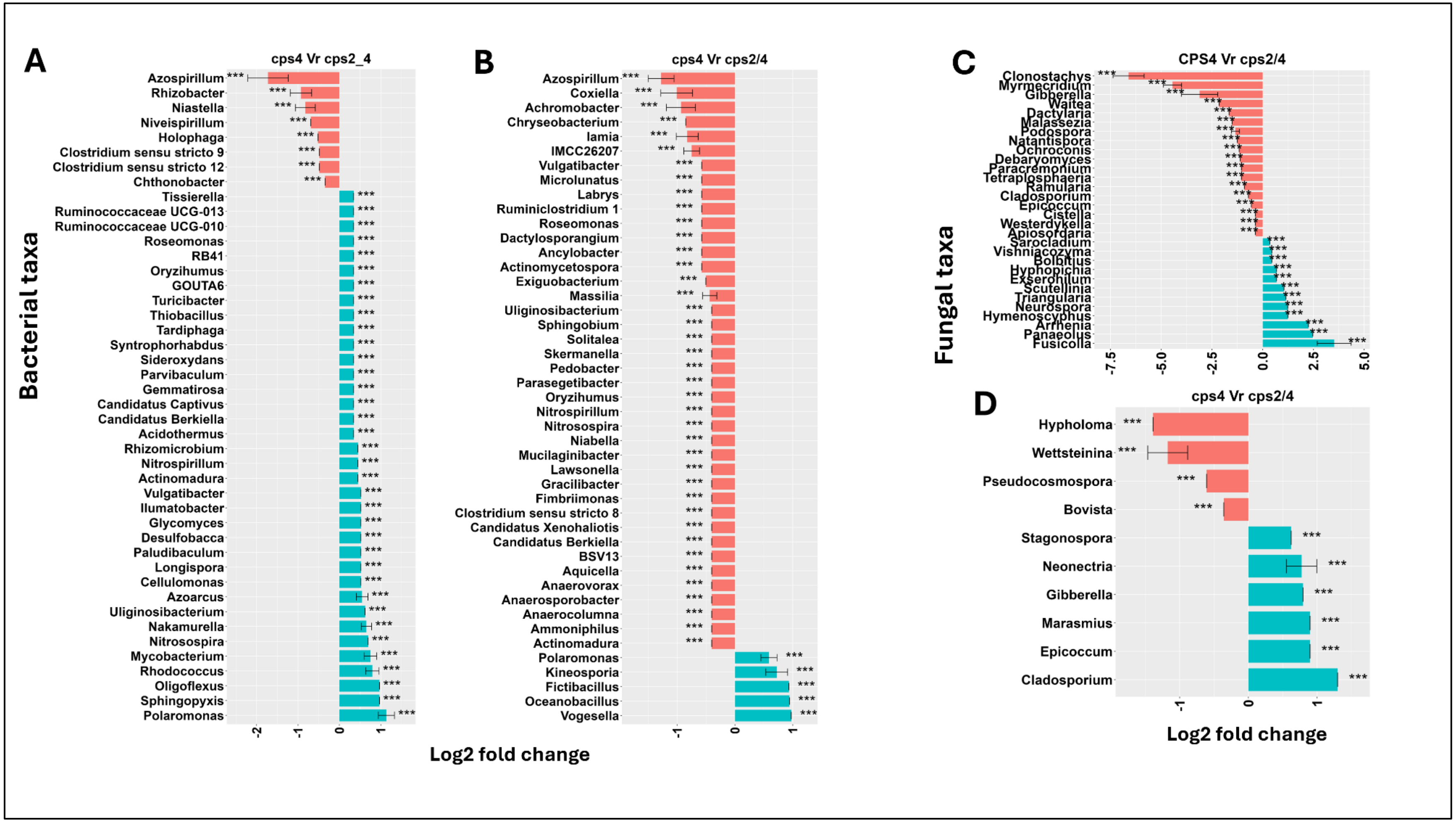

Fig. S9. Differentially abundant (Log2 fold change) root bacterial taxa between cps4 (red) and cps2/4 (green) at A)17dpt and B) 28dpt and fungal genera at C) 17dpt and D) 28dpt. Data are represented by log fold change (shown as a column), ±SE (shown as error bars) derived from the ANCOM-BC model. The significance of test is indicated as ***for $p < 0.001$, ** for $p < 0.01$ and * for $p < 0.05$.

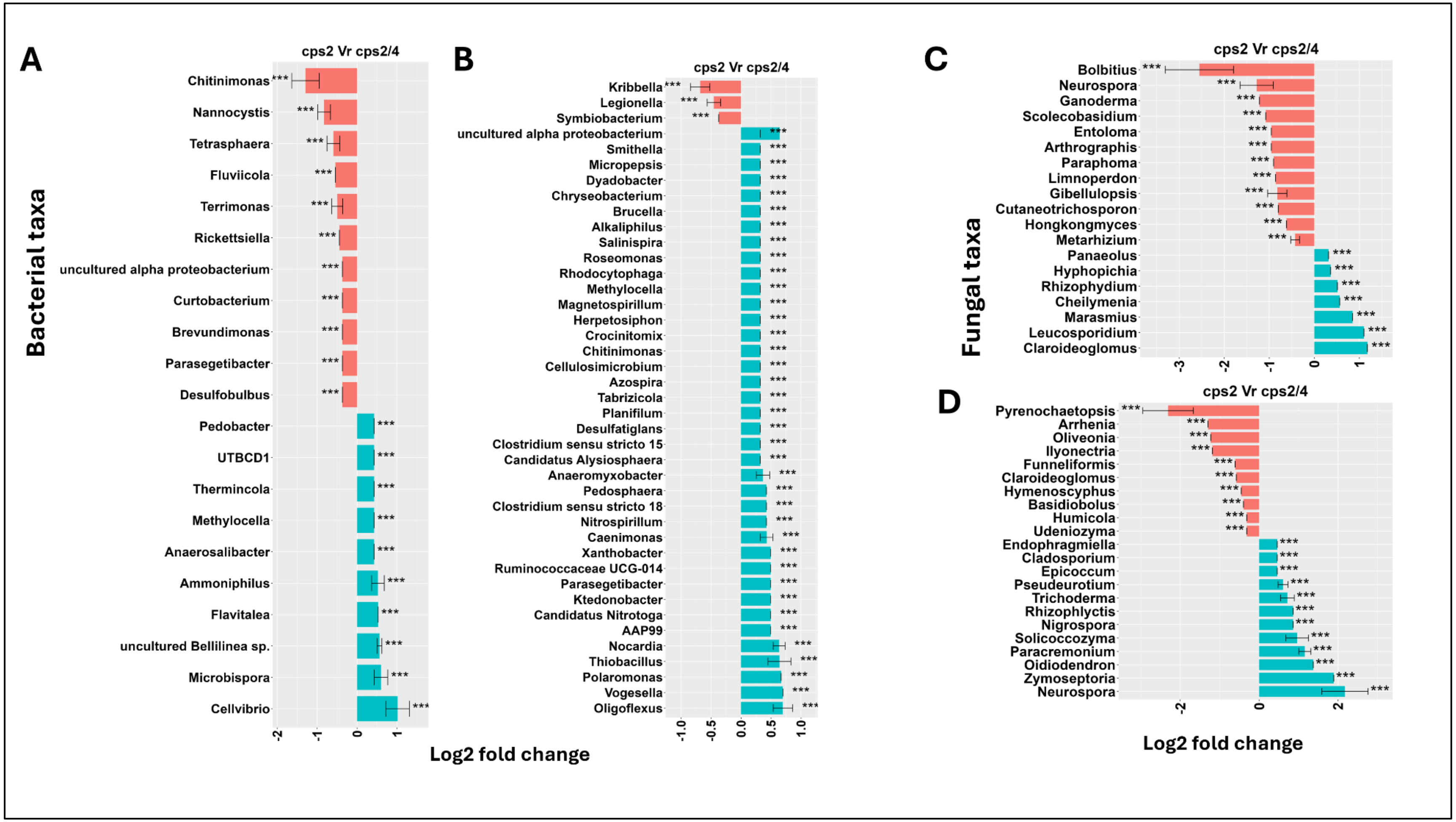

Fig. S10. Differentially abundant (Log2 fold change) rhizosphere bacterial taxa between cps2 (red) and cps2/4 (green) at A)17dpt and B) 28dpt and fungal genera at C) 17dpt and D) 28dpt. Data are represented by log fold change (shown as a column), ±SE (shown as error bars) derived from the ANCOM-BC model. The significance of test is indicated as ***for p < 0.001, ** for p < 0.01 and * for p < 0.05.

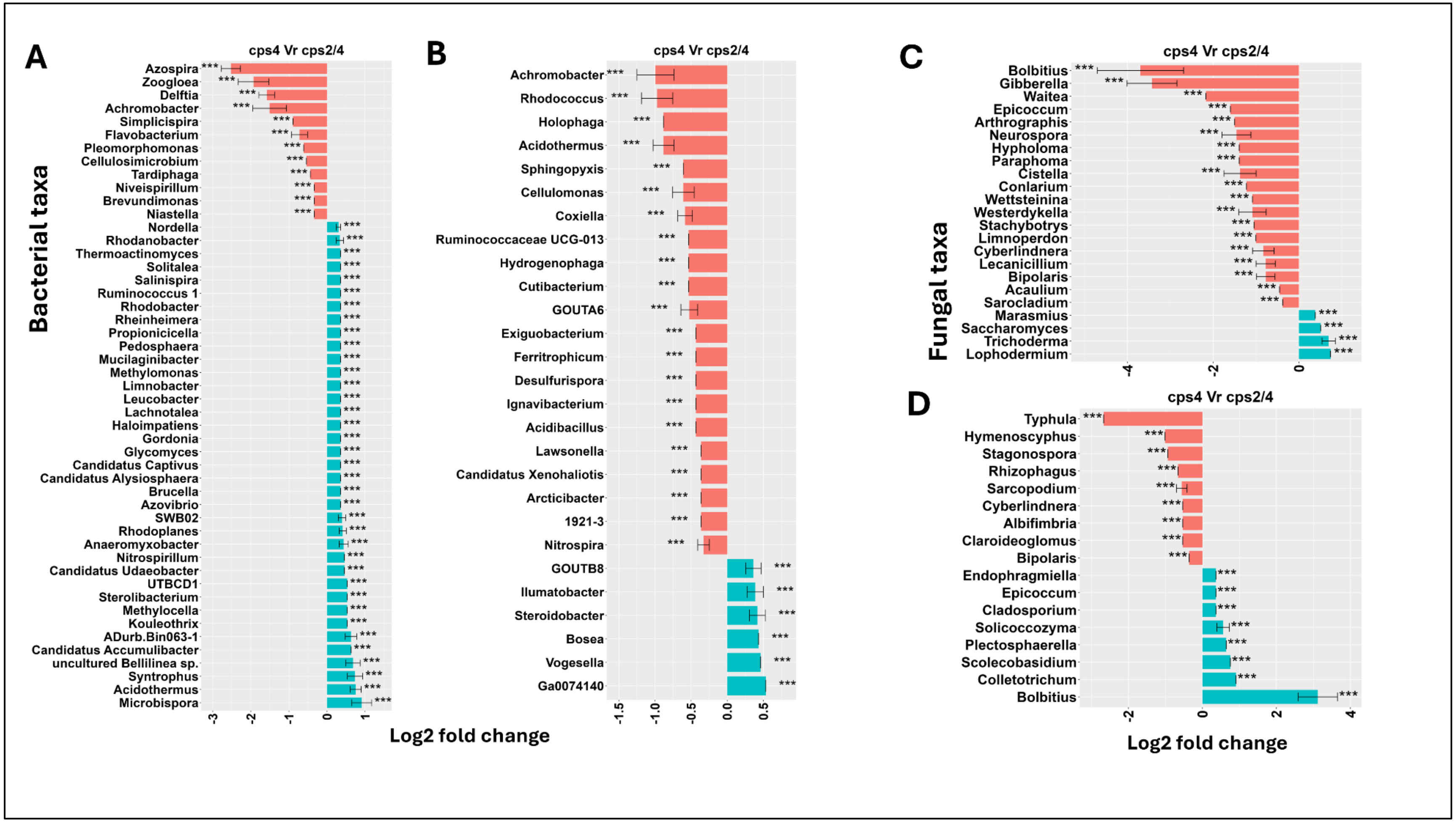

Fig. S11. Differentially abundant (Log2 fold change) rhizosphere bacterial taxa between cps4 (red) and cps2/4 (green) at A)17dpt and B) 28dpt and fungal genera at C) 17dpt and D) 28dpt. Data are represented by log fold change (shown as a column), ±SE (shown as error bars) derived from the ANCOM-BC model. The significance of test is indicated as ***for $p < 0.001$, ** for $p < 0.01$ and * for $p < 0.05$.

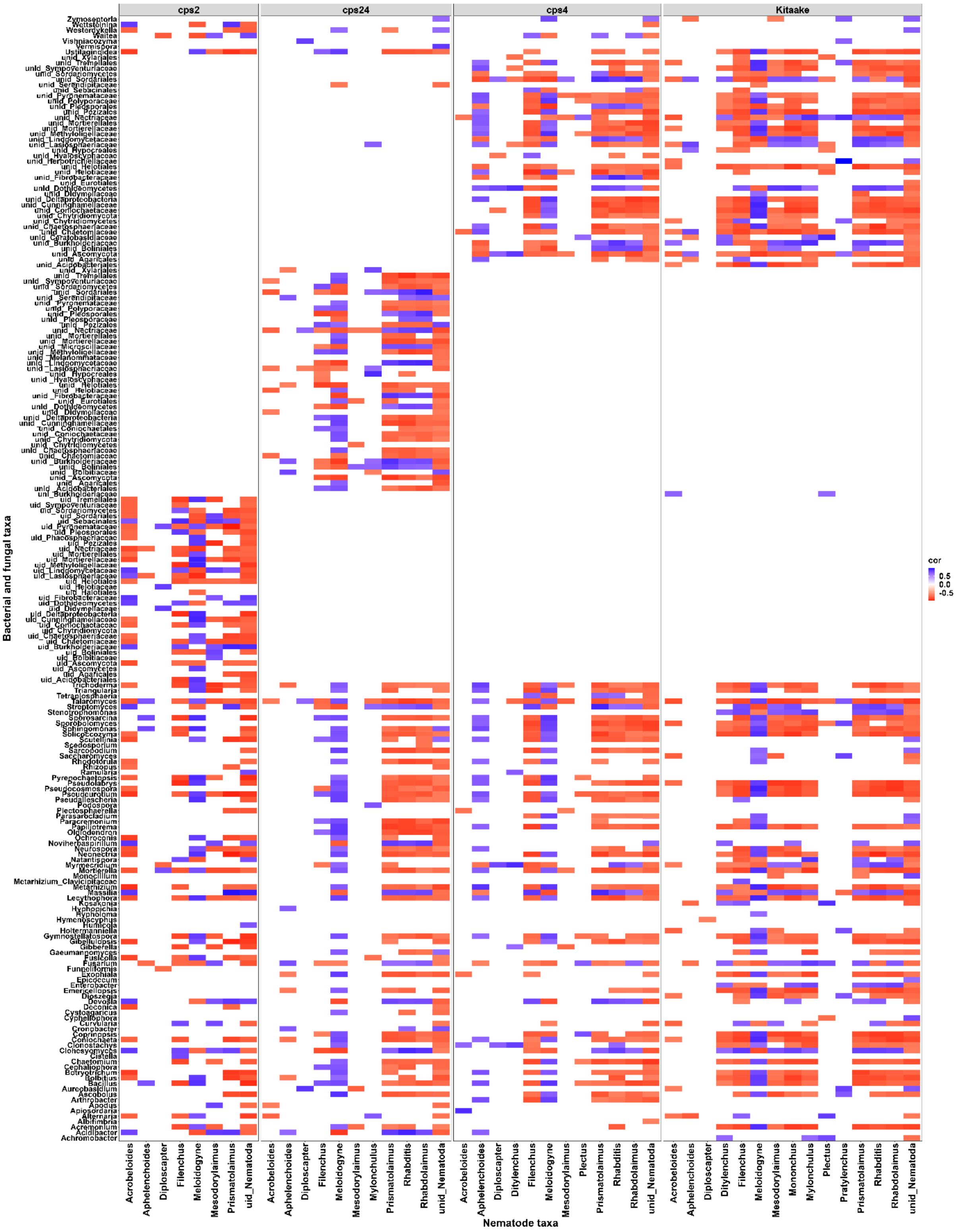

Fig. S12. Heat map of microbial-nematode associations in rice WT and mutants. Both microbes that positively and negatively correlated with *M. graminicola* were used for the visualization.

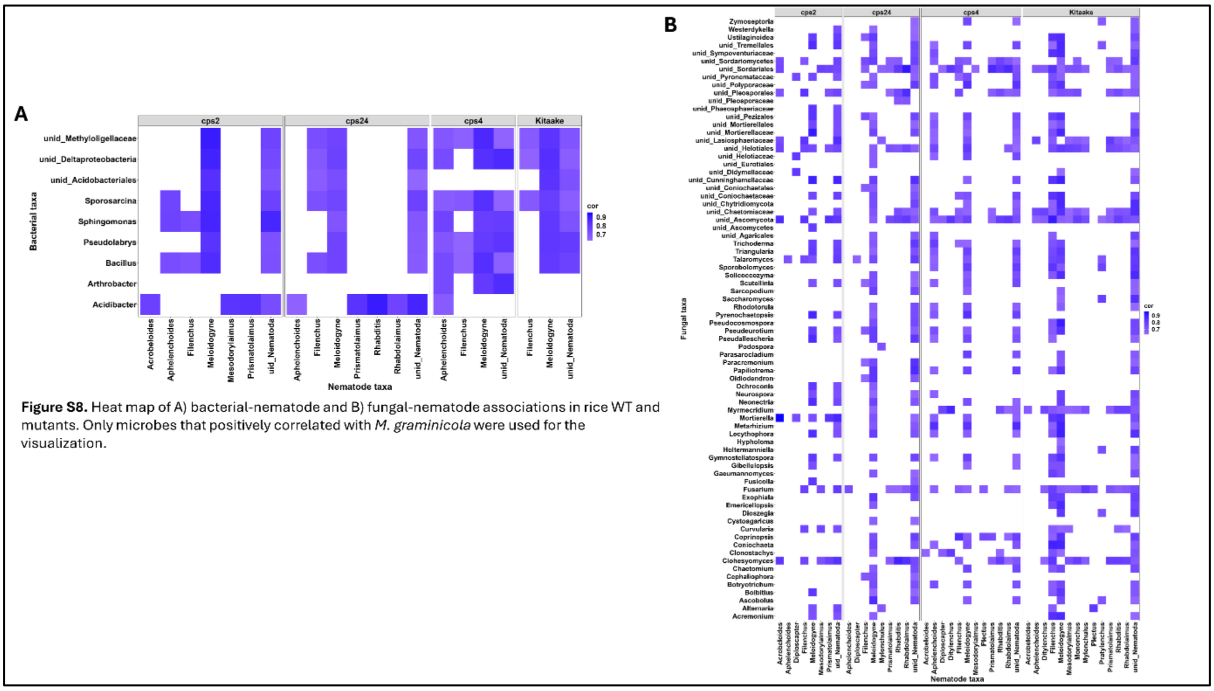

Figure S8. Heat map of A) bacterial-nematode and B) fungal-nematode associations in rice WT and mutants. Only microbes that positively correlated with *M. graminicola* were used for the visualization.

Fig. S13. Figure S13. Heat map of microbial-nematode associations in rice WT and mutants. Only microbes that positively correlated with *M. graminicola* were used for the visualization.

Table S1: Primer sets used in this study for the amplification of bacterial 16S rRNA V5-7 genes. Multiplex identifiers (MID) are indicated in bold in forward primer (799F).

Name	Overhang	MID	Primer
F20-799F	TCG TCG GCA GCG TCA GAT GTG TAT AAG AGA CAG	**CGA GTT**	AAC MGG ATT AGA TAC CCK G
F21-799F	TCG TCG GCA GCG TCA GAT GTG TAT AAG AGA CAG	**GAA CGT**	AAC MGG ATT AGA TAC CCK G
F22-799F	TCG TCG GCA GCG TCA GAT GTG TAT AAG AGA CAG	**GAC TTC**	AAC MGG ATT AGA TAC CCK G
F23-799F	TCG TCG GCA GCG TCA GAT GTG TAT AAG AGA CAG	**GAG TCA**	AAC MGG ATT AGA TAC CCK G
F24-799F	TCG TCG GCA GCG TCA GAT GTG TAT AAG AGA CAG	**GAT GAC**	AAC MGG ATT AGA TAC CCK G
1193R	GTC TCG TGG GCT CGG AGA TGT GTA TAA GAG ACA G		AC GTC ATC CCC ACC TTC C

Table S2: Mean, median and range of reads per compartment for fungal and bacterial libraries at different days post inoculation (DPI).

	Compartment	DPI	Mean	Median	Min	Max	Total
Fungi	Root	17	17496.2	12458.5	4806	50516	349924
		28	13995.75	11901	7436	33160	279915
	Rhizosphere	17	25227.9	21093.5	7477	83684	504558
		28	9100.95	8336	2391	16242	182019
Bacteria	Root	17	4894.125	4831	3265	7515	78306
		28	4994.0625	4824.5	3004	6974	79905
	Rhizosphere	17	7526	7493.5	6815	8458	120416
		28	5660.875	5503.5	5120	6661	90574

Significance of test indicated as ***, p <0.001; **, p <0.01; *, p <0.05. The ns denotes not statistically significant and R^2 is the proportion of variation explained.

Table S3: Permutation analysis of variance (PERMANOVA) using "adonis" test on Bray-Curtis distance matrices for bacterial and fungal community dissimilarity assessment using 1000 permutations

**Community**	**Factor**	**R^2**
Bacteria	Genotype	0.12***
	Compartment	0.57***
	Genotype*Compartment	n.s
Fungal	Genotype	0.11***
	Compartment	0.43***
	Genotype*Compartment	n.s

Significance of test indicated as ***, p <0.001; **, p <0.01; *, p <0.05. The ns denotes not statistically significant and R^2 is the proportion of variation explained.

Table S4: PERMANOVA (Pairwise-adonis) between rice mutants cps2 and cps4

	DPI	Compartment	Factors	R^2
Bacteria	17	root	cps2_vs_ cps4	0.20 ns
			cps2_vs_ cps2_4	0.19 ns
			cps4_vs_ cps2_4	0.18 ns
		rhizosphere	cps2_vs_ cps4	0.16 ns
			cps2_vs_ cps2_4	0.14 ns
			cps4_vs_ cps2_4	0.16 ns
	28	root	cps2_vs_ cps4	0.22ns
			cps2_vs_ cps2_4	0.26 ns
			cps4_vs_ cps2_4	0.17 ns
		rhizosphere	cps2_vs_ cps4	0.18*
			cps2_vs_ cps2_4	0.18*
			cps4_vs_ cps2_4	0.14
Fungal	17	root	cps2_vs_ cps4	0.13 ns
			cps2_vs_ cps2_4	0.26 ns
			cps4_vs_ cps2_4	0.23 ns
		rhizosphere	cps2_vs_ cps4	0.18 ns
			cps2_vs_ cps2_4	0.18 ns
			cps4_vs_ cps2_4	0.19 ns
	28	root	cps2_vs_ cps4	0.17 ns
			cps2_vs_ cps2_4	0.19 *
			cps4_vs_ cps2_4	0.15 ns
		rhizosphere	cps2_vs_ cps4	0.08 ns
			cps2_vs_ cps2_4	0.09 ns
			cps4_vs_ cps2_4	0.14 ns

Significance of test indicated as ***, p <0.001; **, p <0.01; *, p <0.05. The ns denotes not statistically significant and R^2 is the proportion of variation explained.
